# Protein Disorder and Short Conserved Motifs in Disordered Regions Are Enriched near the Cytoplasmic Side of Single-Pass Transmembrane Proteins

**DOI:** 10.1371/journal.pone.0044389

**Published:** 2012-09-04

**Authors:** Ilias Stavropoulos, Nora Khaldi, Norman E. Davey, Kevin O’Brien, Finian Martin, Denis C. Shields

**Affiliations:** 1 School of Medicine and Medical Science, University College Dublin, Dublin, Ireland; 2 Complex and Adaptive Systems Laboratory, University College Dublin, Dublin, Ireland; 3 Conway Institute, University College Dublin, Dublin, Ireland; 4 School of Biomolecular and Biomedical Science, University College Dublin, Dublin, Ireland; 5 Structural and Computational Biology Unit, European Molecular Biology Laboratory, Heidelberg, Germany; 6 Department of Food Science and Technology, University of California Davis, Davis, California, United States of America; University of Toronto, Canada

## Abstract

Intracellular juxtamembrane regions of transmembrane proteins play pivotal roles in cell signalling, mediated by protein-protein interactions. Disordered protein regions, and short conserved motifs within them, are emerging as key determinants of many such interactions. Here, we investigated whether disorder and conserved motifs are enriched in the juxtamembrane area of human single-pass transmembrane proteins. Conserved motifs were defined as short disordered regions that were much more conserved than the adjacent disordered residues. Human single-pass proteins had higher mean disorder in their cytoplasmic segments than their extracellular parts. Some, but not all, of this effect reflected the shorter length of the cytoplasmic tail. A peak of cytoplasmic disorder was seen at around 30 residues from the membrane. We noted a significant increase in the incidence of conserved motifs within the disordered regions at the same location, even after correcting for the extent of disorder. We conclude that elevated disorder within the cytoplasmic tail of many transmembrane proteins is likely to be associated with enrichment for signalling interactions mediated by conserved short motifs.

## Introduction

Reports over the last decade have shown that a defined three-dimensional structure is not always essential for a protein to be functional. In fact the presence of disordered regions have often been implicated in mediating protein function [1], [2]. Disordered regions in some proteins may remain constantly unstructured, but many acquire a defined three-dimensional structure upon binding to a target [3], [4]. Disordered regions are characterized by enrichment in polar and charged amino acids, and by a low proportion of hydrophobic amino acids [5]. Polar and charged amino acids are in general more flexible and more functionally active [6]. The skewed amino acid composition and residue distribution within disordered regions allowed the development of bioinformatics tools that predict disorder [7].

The function of disordered regions often relies on short linear motifs contained therein [8]. In general, disorder is not under strong evolutionary conservation, thus any degree of conservation observed in disordered regions indicates potential functionality importance [9]. Short linear motifs are stretches of typically fewer than ten amino acids in length, usually with less than five defined positions [10]. They often act as recognition sites for protein modification, as protein cleavage sites and as targeting motifs for subcellular localization [11]. Their short length gives a small interface between the motifs and their protein partners, with the affinity of interactions typically weaker than that for typical interactions between globular domains with larger interfaces [12]. These interactions are usually transient, reversible and modulatable, making them ideal for mediating conditional interactions [13], [14]. The compact interfaces and the ubiquity of motif mediated interactions within interaction networks [15] make these interactions amenable to chemical targeting.

A previous study showed that 40% of human transmembrane proteins contain disordered regions [16]. In the same study, it was shown that there is a preference for these disordered regions to reside in the intracellular parts of the proteins, rather than in the extracellular side. While on average single-pass proteins did not seem to share this characteristic, with disordered regions being distributed evenly on both sides of the membrane, this effect was suggested to reflect a small number of single-pass proteins with large extended glycosylated extracellular disordered regions [16]. In the present study, we set out to determine if there is an enrichment of disorder and evolutionarily conserved motifs on the juxtamembrane cytoplasmic side of single-pass transmembrane proteins, which we considered likely to be enriched for signalling interactions; and we identify which regions close to the membrane show such enrichment effects.

## Results

### Distribution of Disorder in Cytoplasmic and Extracellular Segments of Human Single-pass Transmembrane Proteins

A total of 803 single-pass human transmembrane proteins annotated with defined extracellular, transmembrane and intracellular regions, and for which we were able to identify more than six orthologues from the Metazoa kingdom, were used to study the distribution of disorder and motifs in relation to the plasma membrane. The original positions of all extracellular and intracellular residues were mapped onto a uniform scale by defining in each case as first position the first residue adjacent to the membrane on the extracellular or intracellular side respectively. The disorder scores for each position on the new scale were calculated by averaging all the disorder scores that fall in the same position.

**Figure 1:**
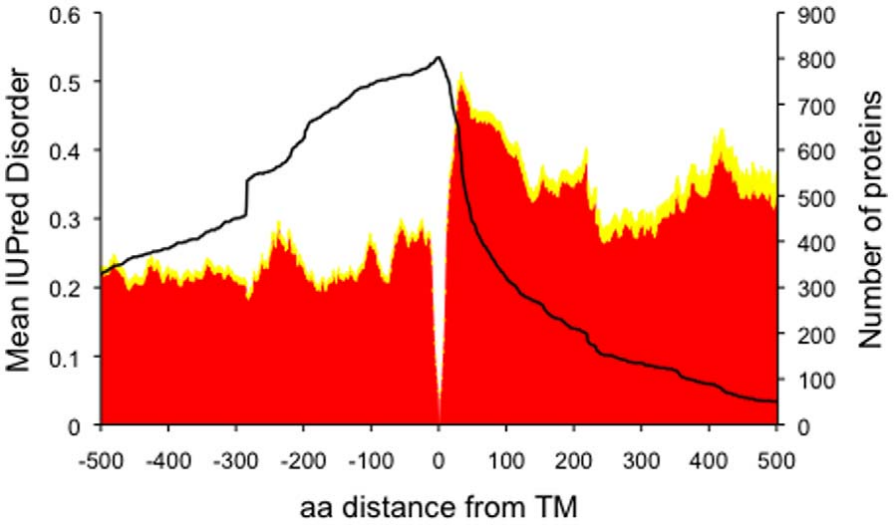
Disorder profile of single-pass proteins. Mean IUPred disorder profile (in red) for residues from 803 human single-pass transmembrane proteins, with respect to distance from the membrane (position 0; extracellular negative, intracellular positive). The membrane region was included in the analysis, but has been omitted from the graph. Standard errors are displayed in yellow. The number of proteins that have a residue at a specific position is shown with a black line. TM: transmembrane.

Figure 1 shows that the average disorder is greater in the cytoplasmic tails of the single-pass proteins than in the extracellular side. It is known that in human transmembrane proteins disordered regions are preferentially localized in the cytoplasmic tails and are sparse in the extracellular segments [16]. Although single-pass proteins in particular were previously noted to have a similar distribution of disordered regions on both sides [16], in the present study the cytoplasmic tails appear to be more disordered.

In Figure 1, there is an observed intracellular peak in disorder 30 amino acids from the membrane. A highly structured region is observed around the plasma membrane (Figure 1), which is not surprising given the highly hydrophobic and overwhelmingly alpha-helical structure present in the human plasma membrane. The regions after position 30 are characterized by a decrease in disorder. The difference between regions appears significant, since the mean disorder is 0.491 around position 30 (standard error of 0.008), while it is 0.445 around position 60 (standard error of 0.012).

We were concerned that this embedded ordered region within the sequence could bias the results seen at a certain distance from the membrane, since IUPred [7] calculations incorporates long-range potential energy interactions. To ensure that the observed distribution was robust, the membrane regions of the proteins were removed and disorder was predicted again. Unsurprisingly, the previously observed structured region around the membrane disappears entirelly when the membrane region was not included in the disorder prediction (Figure S1). However, apart from this difference, the overall findings are similar, including both the higher average disorder in the cytoplasmic segments than the extracellular, and the peak in disorder at 30 residues from the membrane. Thus, the observed disorder patterns are not merely a computational artifact of the adjacency of the hydrophobic transmembrane region.

**Figure 2:**
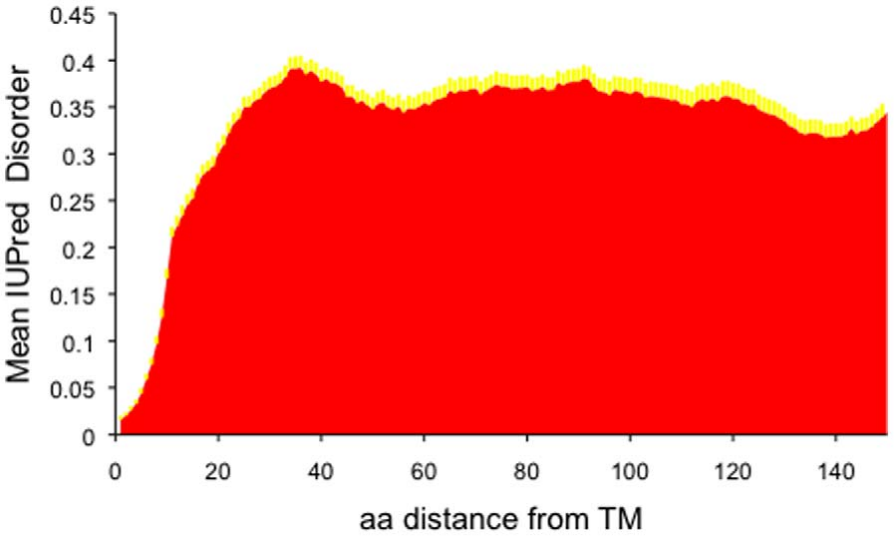
Disorder profile of the cytoplasmic parts of 263 single-pass transmembrane proteins with long cytoplasmic tails. (>150 residues). Standard errors of the mean values are displayed in yellow.

A noteworthy property of single-pass proteins is that they have shorter cytoplasmic and longer extracellular tails. Approximately 65% of the proteins have cytoplasmic tails of 150 residues long or shorter (Figure 1). This has implications for interpretation for disorder, since shorter sequences are typically more disordered, with disorder increasing near the terminus. Thus, the pattern of increased disorder around residue 30 might simply have arisen as a consequence of a biased distribution in cytoplasmic tail lengths, rather than being an inherent property of the sequences. As such, we chose the subset of sequences whose cytoplasmic tails exceeded 150 residues in length, accordingly. In Figure 2 only proteins with long tails are shown. In this case the disorder still peaks at around position 35 with a score of 0.391 (standard error of 0.012), which is higher than seen at position 60, with a disorder score of 0.353 (standard error of 0.013). However, the pattern is less striking.

### Distribution of Predicted Short Linear Motifs in Disordered Regions of Human Single-pass Proteins

Motifs strongly prefer to reside inside locally unstructured regions [1], [17]. We sought, therefore, regions in the proteins that are characterized simultaneously by high intrinsic disorder and increased relative local conservation in orthologous proteins. Although motifs are not characterized by the same strong evolutionary constraints as globular domains, functional motifs are relatively conserved compared to their adjacent residues [9]. This conservation is often clearly visible in multiple sequence alignments of known functional motifs as islands of constraint in otherwise rapidly evolving regions. Here, we identified putative functional motifs by searching for short linear motif “fingerprints”, regions of relative conservation in disordered regions, indicative of a functional role. To estimate the Relative Local Conservation (RLC) of a disordered residue, we compared its conservation to the background distribution of adjacent disordered residues within the sequence, calculating the probability of its level of relative conservation. We then calculated the likelihood of several over-conserved residues occurring in close proximity by chance, allowing up to five residues in a “motif” with a maximum wildcard length of 2 between any two defined residues. Using this method, all human single-pass protein sequences were searched for statistically over-conserved groupings of residues.

The intracellular tails of the 803 single-pass proteins were predicted to have 411 conserved motifs (2–13 residues in length, with mean length 7.8, and standard deviation 1.9). The extracellular tails were predicted to contain 393 conserved motifs (3–13 residues in length, with mean 8.21 and standard deviation 1.9). Although the extracellular tails appear less abundant in disorder, both extracellular and intracellular tails seem to contain similar numbers of predicted conserved motifs in disordered regions, since the extracellular tails are correspondingly larger.

**Figure 3:**
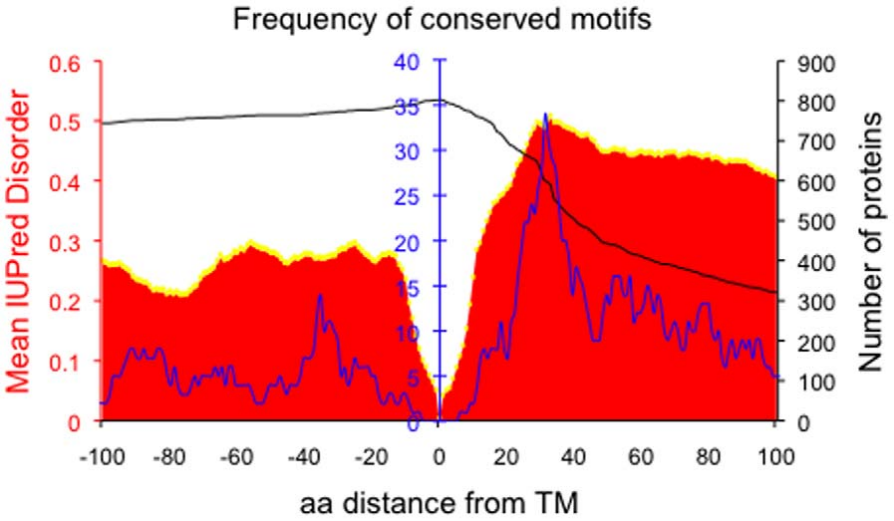
Frequency of conserved motifs. The frequency (blue line) depicts the number of instances that a specific residue position falls inside a predicted motif. Disorder profile of single-pass transmembrane proteins when the membrane region was included in the analysis is shown in red, with standard errors displayed in yellow. The number of proteins that have a residue at a specific position is shown with a black line.

**Figure 4:**
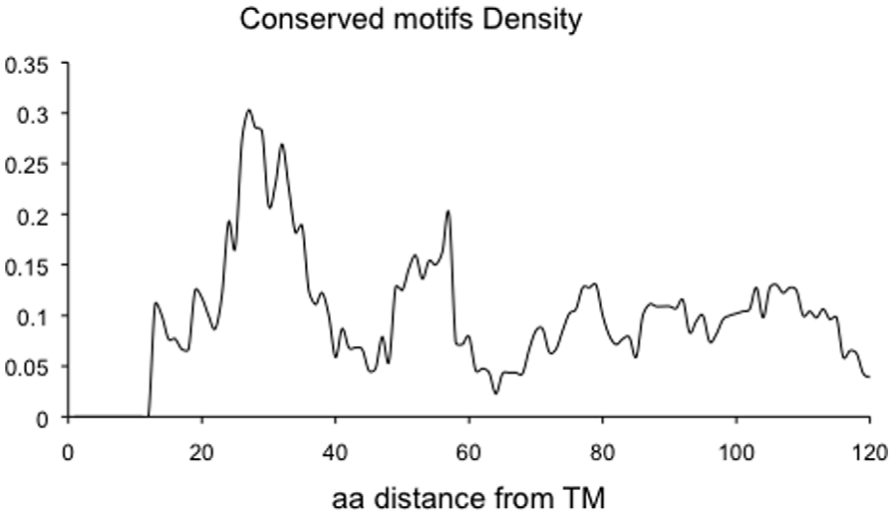
Conserved motif density in long (>150 residues) cytoplasmic tails of single-pass transmembrane. Conserved motifs density is defined as the number of conserved motifs that span a residue divided by the number of residues that are disordered.

Figure 3 shows the frequency of predicted conserved motifs across the proteins in relation to the plasma membrane. A marked accumulation of conserved intracellular motifs is observed around 30 amino acids from the membrane, in the same position where the increased disorder was noted (Figure 3). The dataset is not non-redundant, including members of proteins families in the analysis. However we do not think this has strongly biased the results. Firstly, the prediction of motifs depends on the conservation of a motif among orthologous proteins and not among paralogues. Secondly, while over-enrichment of a few families could potentially have accounted for the enrichment of motifs around 30 residues from the membrane, Table S3 indicates that the motifs are drawn from a wide variety of proteins. The peak in predicted motifs is present regardless of the presence or absence of the membrane region during the analysis (data not shown). Moreover, when the motif density was investigated in proteins with long cytoplasmic tails (greater than 150 residues), a similar pattern was observed, although the smaller sample size made it statistically less significant. Nevertheless, we noted that the first 60 residues closest to the membrane had a significantly higher frequency of motifs compared to the following 60 residues (p-value = 0.02, ANOVA test; Figure 4).

**Figure 5:**
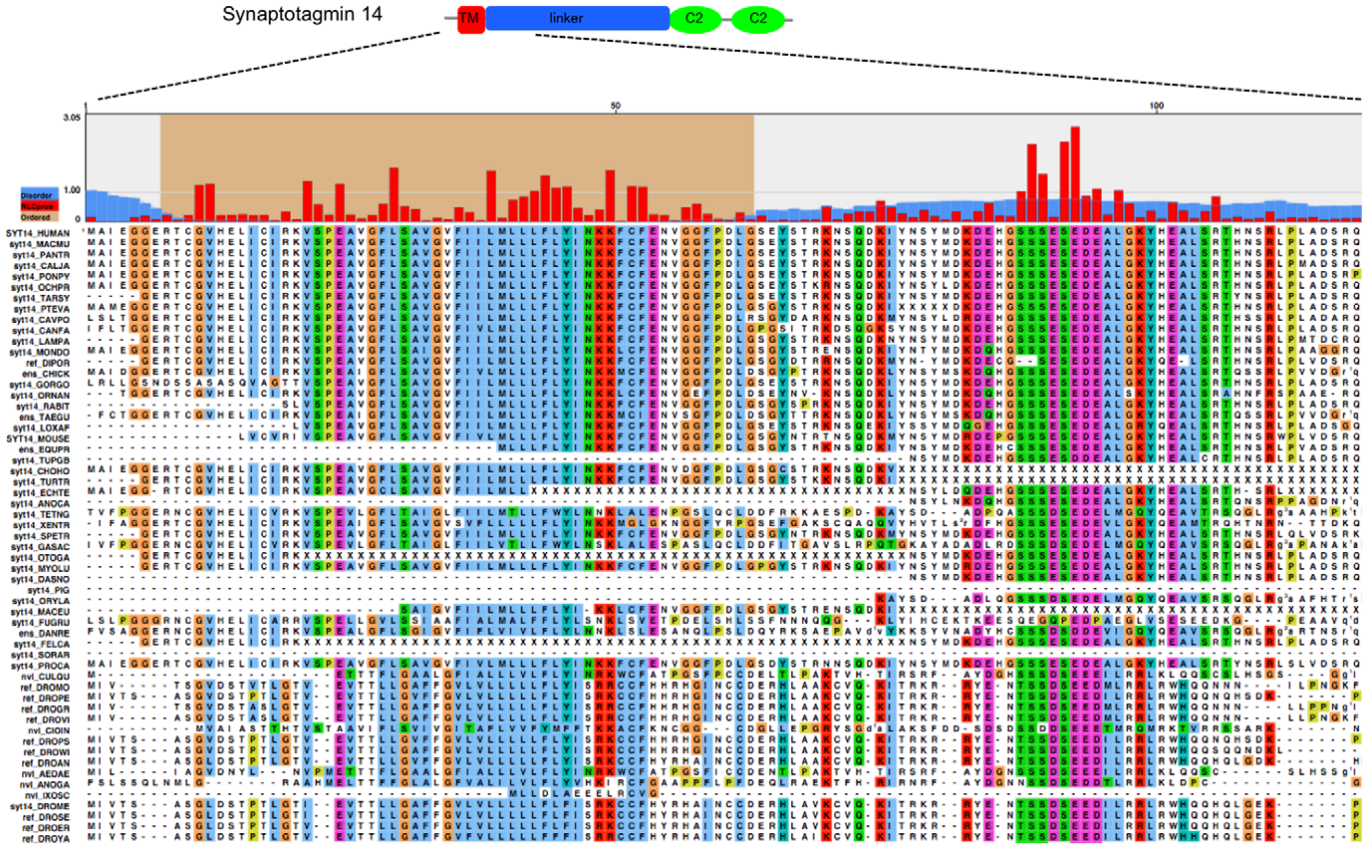
Evolutionarily conserved motif region in the Synaptotagmin 14 linker region. The upper panel shows a graphical representation of whole Synaptotagmin 14 protein. The transmembrane region (TM), the linker and the two C2 domains are shown. The lower panel shows the first 120 residues of an alignment of synaptotagmin 14 with identified orthologues. Red bars indicate Relative Local Conservation (RLC) and the blue bars indicate IUPred disorder scores. The brown background shows an ordered part (IUPred score ><0.3) around the transmembrane region.

A list of the most significant conserved motifs predicted in the present study is shown in Table S1. An example of a conserved cytoplasmic tail motif predicted in Synaptotagmin-14 is shown in Figure 5. Synaptotagmins form a wide family of putative membrane trafficking proteins present in various species from humans to plants [18]. The presence of synaptotagmins in various cell types and organisms suggests that they have evolved varying functions [19], including calcium dependent synaptic vesicle exocytosis in neurons [20]. The identified motif lies in the charged “linker” regions between the membrane and the conserved cytoplasmic C2 domain, which is responsible for binding to phospholipids on the vesicles in a calcium dependent or independent manner [21]. The linker region is generally fairly unconserved, but the residues of the predicted motif SSxSExE are conserved in all synaptotagmin-14 orthologues from mammals to fish and to insects (Figure 5), suggesting a potential functional relevance of this region. This motif has not been previously identified. We note that it is a potential casein kinase II substrate, based on similarity to a known motif for this enzyme (Table S1), so its function may well depend on its phosphorylation state. Casein kinase II has been shown to phosphorylate the bovine synaptotagmin I in a single Threonine in the linker region, playing a role on the nerve terminal function [22]. It is possible that one of the Serines located in the predicted conserved motif serve as a phosphorylation site for a kinase. The accumulation of Lysine, Aspartic acid and Glutamic acid in the surrounding regions also corroborates this possibility [22]. It is possible that phosphorylation induces structure formation in the disorder linker as has been seen in other cases [23], [24]. Such transient structure formation upon phosphorylation and under the regulation of Ca2+ levels potentially facilitates the interaction of synaptotagmin with the phospholipid membrane during synaptic vesicle exocytosis [22]. Cytoplasmic tails of transmembrane proteins are often casein kinase II substrates during protein trafficking from the Golgi to the membrane or to the lysosomes [25], [26], [27]. The method clearly needs sufficient evolutionary change in order to detect a signal, but not so much that the signal is wiped out. In our analyses, the most significant motifs were often conserved across to non-vertebrate groups, such as flies. As can be seen in Figure 5, the residues highlighted in the SSxSExE motif of Synaptotagmin-14 within the motif are either completely conserved in flies or, in the case of the last residue, are replaced by a highly similar amino acid (D). In contrast, the flanking disordered region residues (Fig. 5, from SEYSTR onwards) show considerable evolutionary differences in D.melanogaster, with 24 dissimilar residues, and only 5 identical residues. For more rapidly evolving proteins, there is sufficient evolutionary change in the flanks over shorter evolutionary spans to provide a signal to detect motifs.

Other motifs bear partial or striking similarity to known motifs (Table S1), which we identified using the CompariMotif program [28], drawing on known motif databases including ELM [8] and MiniMotif [29]. For instance the motif predicted on the juxtamembrane region of low-density lipoprotein receptor-related protein 4 (LRP4) LTxxNPxY (Table S1) contains the endocytic motif NPxY. The endocytic motif was initially identified within the cytoplasmic tail of LDLR, where it serves as a signal for receptor endocytosis through coated vesicles [30], [31]. Rapid internalization of the cell surface LDL receptor strongly depends on this motif [30].

The distribution of experimentally proven motifs in the ELM database (Figure S2) in cytoplasmic tails of more than 150 residues did not show the same juxtamembrane preference, with only a few instances of binding motifs and modification motifs in that region. While experimental biases relating to technical ease of evaluating membrane proximal regions, or biological differences in the classes of motifs identified here and by ELM, could account for the differences in findings, the sample size from ELM is too small to draw any strong conclusions.

### Amino Acid Composition

**Figure 6:**
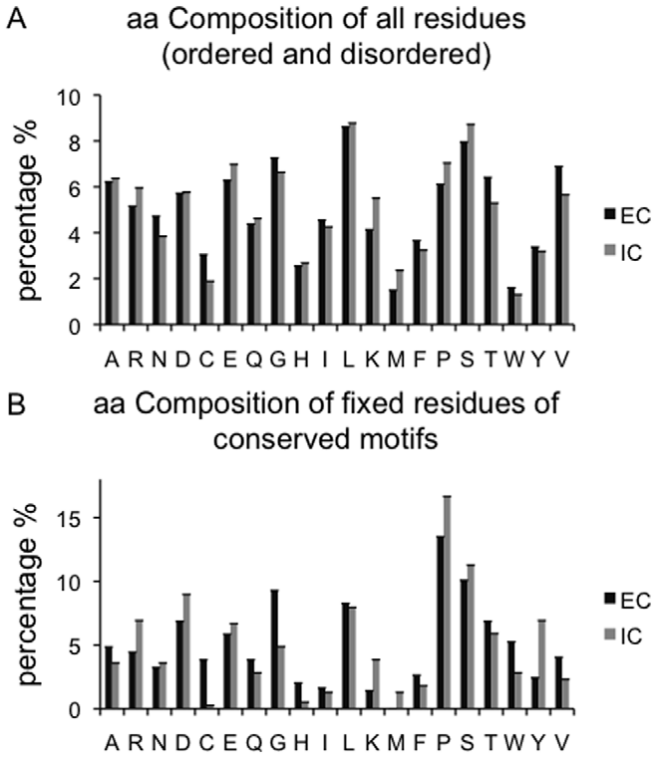
Amino acid composition. Standard error bars plotted on the graph are very close to the mean values. EC: extracellular. IC: intracellular.

The amino acid compositions of extracellular and intracellular regions of the single-pass proteins were analyzed, considering only extracellular and intracellular tails of greater than 150 residues (Figure 6 and Figure S3). As expected, disordered regions are depleted in hydrophobic order-promoting amino acids such as W, L, F and Y. No significant differences were observed when the composition of disordered residues in the extracellular segments was compared to that of the intracellular parts, apart from the case of Threonine and Serine (Figure S3). Threonine comprises 9.2% of extracellular disordered residues, compared with only 5.8% of intracellular. In contrast, Serine is more abundant in the intracellular disordered residues (8.7% extracellularly and 11.8% intracellularly).

On both sides of the membrane, the predicted motifs are enriched in disorder promoting residues like Proline and Serine (Figure 6B). Fixed residues of intracellular motifs were enriched in Tyrosine (7.0%), compared with only 2.4% of extracellular fixed motif residues. This may relate to intracellular Tyrosine phosphorylation, since many intracellular Tyrosines in disordered regions are phosphorylated. In contrast, Tryptophan is more prevalent in extracellular motifs, perhaps consistent with its role in motifs that are glycosylated. These two findings suggest that a reasonable proportion of motifs may be post-translationally modified in both compartments.

## Discussion

The present study indicates that in human single-pass transmembrane proteins, disordered residues are preferentially found in the cytoplasmic tails and are sparser in the extracellular segments. This cytoplasmic preference of disorder has been previously reported in Type I single-pass transmembrane proteins [32]. In another study, single-pass proteins appear to have an even distribution of disorder in both sides of the membrane when disordered regions of at least 30 consecutive disordered residues are considered [16]. The variable length of disorder and its ability to confer function regardless of its length [1] justified our choice to include disordered regions of any length in our study.

The observed peak of disorder approximately - 30 amino acids from the membrane intracellularly - suggests that the region may be used for the positioning of disordered residues involved in protein-protein interaction and signal transduction. Most of the single-pass proteins are protein receptors and therefore the regions proximal to the membrane are crucial for the transduction of signal upon ligand binding to the extracellular part of the receptor. However, it is difficult to infer that the pattern of disorder is favoured by evolution, since the alternative hypothesis could just as easily be formulated: that the intracellular juxtamembrane region is selectively unimportant for the placement of important ordered functional residues, and is over-represented by unimportant disordered residues. Inspection of the patterns of evolutionary conservation of putative functional motifs in the disordered regions provides some insights into this issue.

We found that the peak of conserved disordered motifs corresponded to the peak of disorder in the intracellular juxtamembrane region (at a distance of 30–35 amino acids). This suggests that this area is favoured by selection for motifs. Since the disorder peak also occurs in the same area, we hypothesise that the peak in disorder may also be selectively advantageous.

Although the extracellular segments were predicted to have low average disorder, they still have a large number of predicted motifs; this suggests that proteins exploit even limited disordered regions to implant potentially functional motifs. Just a few residues inside a disordered sequence are able to support protein interactions [17]. Motifs in extracellular tails may often facilitate post-translational modifications, such as glycosylation, protecting the disordered regions from cleavage. This notion is also supported by the enrichment of Tryptophan (W) in extracellular motifs, since this residue may be modified by glycosylation (Figure 6B). Post-translational modification of motifs provides a potential mechanism whereby motifs may be activated or inactivated in a regulated fashion. Judging by the amino acid composition of fixed residues of the predicted conserved motifs (Figure 6B), it is clear that the motifs are largely similar on both sides of the membrane. The main differences are that Glycine is more prevalent in the extracellular motifs, with Tyrosine and Proline more common in the intracellular. The prevalence of Glycine in extracellular motifs may suggest a potential role in flexible regions conferring mobility on the extracellular terminus of the protein. The observed enrichment of Tyrosine (Y) in intracellular motifs is consistent with the importance of Tyrosine phosphorylation and de-phosphorylation in many signalling events.

Disordered regions are not generally under such strong evolutionary constraints as structured regions, thus their conservation in that area implies their importance, potentially due to their functional activity. Most signal transduction events involve binding of a ligand to the extracellular part of the receptor with the cytoplasmic tail being responsible for conveying the signal intracellularly by interacting with target proteins inside the cell. The short length of motifs makes them ideal for forming transient interactions, which need to be specific while being readily formed and disrupted [15]. Functional instances of short disordered motifs have been shown previously to be involved in many signalling pathways [33]. We hypothesize that the enrichment of conserved disordered motifs in the juxtamembrane region of single-pass proteins has been evolutionary favoured by selection for regions that can take part in juxtamembrane signalling events. The relative ease with which evolution can generate novel short motifs, through a small number of amino acid replacements, may in part explain their widespread utilisation in vertebrate signalling proteins [12].

## Materials and Methods

### Datasets

As the aim was to study the distribution of disorder and motifs in relation to the membrane in single-pass transmembrane proteins, we decided to focus on a fairly well curated dataset. As such, we used the identification of membrane proteins and the assignment of the location and topology as provided in the Uniprot database (http://www.uniprot.org/). Therefore, only proteins annotated to have defined extracellular, transmembrane and cytoplasmic regions, and possess only one of each were selected (803 proteins, selected using the keywords ‘transmembrane’ and ‘Homo sapiens’). Accession numbers for the 803 proteins are provided in the Table S2.

### Identification of Orthologues

The Gopher algorithm [34] was utilised for the identification of orthologous proteins for each of the 803 protein sequences. The algorithm used BLASTP [35], [36] to identify orthologues in metazoan proteins contained in the EnsEMBL sequence database of sequenced genomes. For proteins that had more than six identified orthologues, Gopher generated alignments using Muscle [37]. The alignments of orthologous proteins were used for the calculation of the Relative Local Conservation (RLC) score [11]. IUPred was used to estimate the disorder score for each residue [6], [7].

SLiMPrints combined information from RLC calculation and disorder prediction to identify short conserved motifs in the proteins (Davey et al, in preparation; server to calculate these statistics available at bioware.ucd.ie; for details of the calculations of statistics used in this analysis see [38] and Methods S1). Residues in short motifs are typically under weaker evolutionary constraints compared to globular domains [9], [10]. As such in order to identify conserved regions of disordered proteins, it is important to calculate the conservation relative to surrounding disordered residues of the same protein accordingly [11]. As defined in [11], the RLC considers the similarity of the residue *i* to related proteins (here limited to orthologues) relative to the similarity seen for adjacent residues as RLC_i_ = (C_i_-b_i_/s_i_) where C_i_ is the conservation of the residue, and b_i_ is the mean score across a window of 30 residues to either side of residues with an IUPRED score of greater 0.2, and si is the standard deviation of the RLC values across that window. Only residues with an IUPRED score in excess of 0.5 were considered for inclusion in a SLiMPrint motif. The probability of RLC scores from a background distribution were estimated, and the probability of a run of unlikely RLC scores comprising a motif were then estimated from these scores, in each case by drawing from the background distribution of scores in the dataset (Methods S1). All background residues included for comparison were equally weighted. Two key parameters in determining this motif probability were the maximum number of fixed residues (5 residues), and the maximum gap length between non-wildcard residues (2 residues). This choice of parameters was chosen in part based on our interpretation of the typical features of short linear motifs, as described in the ELM database for example [39], and in part by our desire to restrict the search space to shorter motifs, since the search space of longer motifs is considerably greater, leading to a potential increase in the proportion of false positives to true positives as the search space expands. The maximum motif size is therefore 13 (e.g. RxxRxxRxxRxxR). While two residues do not typically constitute a particularly interesting motif on their own, frequently only a subset of residues in a motif are strongly conserved in evolution, and therefore we allowed motifs with as few as two conserved residues to be considered.

## Supporting Information

Figure S1 **Mean IUPred disorder profile (in red) for residues from 803 human single-pass transmembrane proteins, with respect to distance from the membrane.** (position 0; extracellular negative, intracellular positive). The membrane region was not included in the analysis, and has been omitted from the graph. Standard errors are displayed in yellow. The number of proteins that have a residue at a specific position is shown with a black line. TM: transmembrane.
(TIFF)

Figure S2 **Distribution of experimentally characterized motifs from the Eukaryotic Linear Motif (ELM) database on the intracellular regions of transmembrane proteins.** Only proteins with intracellular regions greater than 150 amino acids in length were used.
(TIFF)

Figure S3 **Amino acid composition of disordered residues.**
(TIFF)

Table S1 **Conserved motifs in the cytoplasmic tails of single-pass transmembrane proteins.** A conservative threshold of 1E-04 was used.
(DOC)

Table S2 **Protein dataset used in the present study.**
(DOC)

Table S3 **List of proteins with predicted conserved motif in the juxtamembrane region.**
(DOC)

Methods S1 **Equations used in the calculation of Relative Local Conservation and the estimation of motif probability.**
(DOC)
