# Supplementary figures and images for: Protein Disorder and Short Conserved Motifs in Disordered Regions Are Enriched near the Cytoplasmic Side of Single-Pass Transmembrane Proteins
Source: PLoS One. 2012 Sep 4;7(9):e44389. doi: 10.1371/journal.pone.0044389 (PMC3433447; doi:10.1371/journal.pone.0044389)

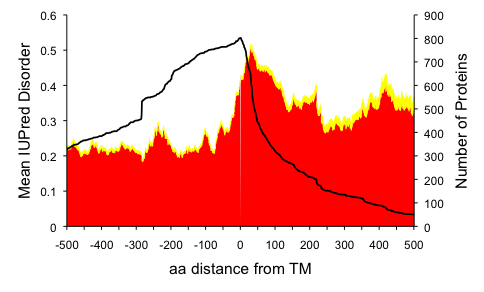

Supplement: Figure S1 — Mean IUPred disorder profile (in red) for residues from 803 human single-pass transmembrane proteins, with respect to distance from the membrane. (position 0; extracellular negative, intracellular positive). The membrane region was not included in the analysis, and has been omitted from the graph. Standard errors are displayed in yellow. The number of proteins that have a residue at a specific position is shown with a black line. TM: transmembrane. (TIFF) [file pone.0044389.s001.tiff]

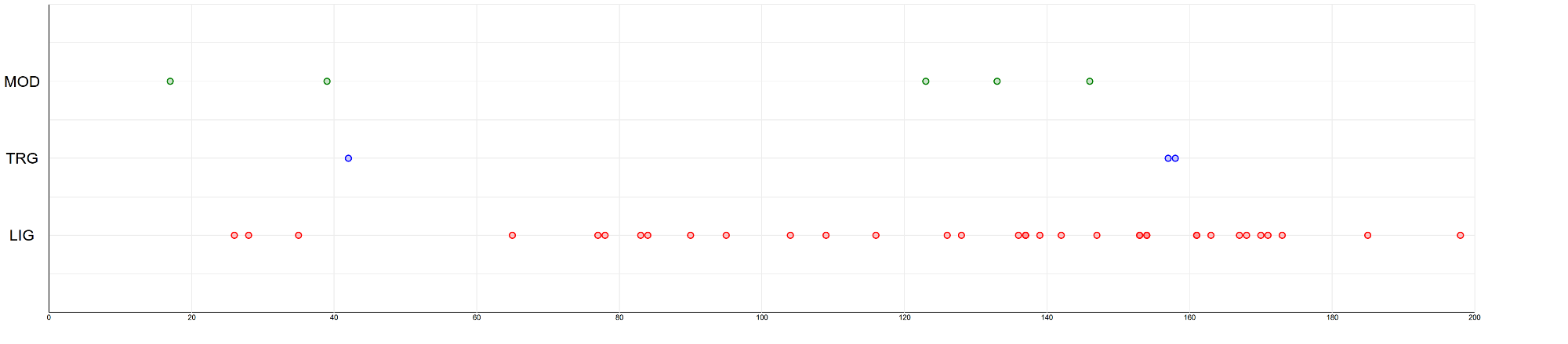

Supplement: Figure S2 — Distribution of experimentally characterized motifs from the Eukaryotic Linear Motif (ELM) database on the intracellular regions of transmembrane proteins. Only proteins with intracellular regions greater than 150 amino acids in length were used. (TIFF) [file pone.0044389.s002.tiff]

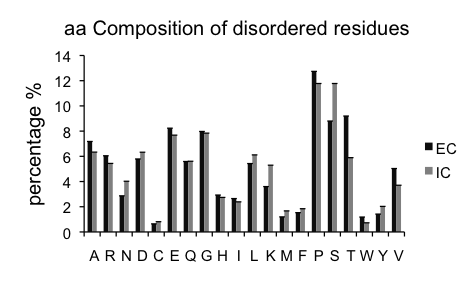

Supplement: Figure S3 — Amino acid composition of disordered residues. (TIFF) [file pone.0044389.s003.tiff]
