# Supplementary material for: Protein Disorder and Short Conserved Motifs in Disordered Regions Are Enriched near the Cytoplasmic Side of Single-Pass Transmembrane Proteins
Source: PLoS One. 2012 Sep 4;7(9):e44389. doi: 10.1371/journal.pone.0044389 (PMC3433447; doi:10.1371/journal.pone.0044389)
Supplement: Table S1 — Conserved motifs in the cytoplasmic tails of single-pass transmembrane proteins. A conservative threshold of 1E-04 was used. (DOC) [file pone.0044389.s004.doc]

| **ID** | **Name** | **RE** | **Prob** | **Sig** | **motif start** | **distance from TM** | **tail-length** | **Similar motifs known**  **Motif name, motif, reference** |
| --- | --- | --- | --- | --- | --- | --- | --- | --- |
| P42658 | DPP6, Dipeptidyl aminopeptidase-like protein 6 | G..PEED | 2E-14 | 3E-10 | 30 | 59 | 94 | LIG_TRAF2, [PSAT].[QE]E, [31]  LIG_SH3, P..DY [32] |
| Q13443 | ADAM9, Disintegrin and metalloproteinase domain-containing protein 9 | P.H..PV.P | 6E-11 | 6E-07 | 754 | 36 | 100 | LIG_SH3, P..P.[KR], [33] |
| Q9UKF5 | ADA29, Disintegrin and metalloproteinase domain-containing protein 29 | S..P..P..S.P | 3E-10 | 3E-06 | 777 | 82 | 124 | LIG_WW, PPLP [34]  LIG_SH3, P..P.[KR], [33] |
| Q8TC27 | ADA32, Disintegrin and metalloproteinase domain-containing protein 32 | E.SS..DT | 4E-10 | 3E-06 | 751 | 48 | 83 |  |
| Q9UKQ2 | ADA28, Disintegrin and metalloproteinase domain-containing protein 28 | P.E..EPP | 5E-10 | 5E-06 | 738 | 52 | 88 | LIG_TRAF6, ..P.E..[FYWHDE]. ,[35] |
| Q8NB59 | SYT14, Synaptotagmin-14 | SS.SE.E | 9E-10 | 2E-05 | 88 | 41 | 507 | Casein kinase II substrate, S..S, [36] |
| Q96RL6 | SIG11, Sialic acid-binding Ig-like lectin 11 | AA..D.PS | 2E-09 | 4E-05 | 584 | 12 | 113 |  |
| Q16832 | DDR2, Discoidin domain-containing receptor 2 | PL..DY..P | 4E-09 | 5E-05 | 476 | 55 | 433 | LIG_SH2, Y..P, [37] |
| P16284 | PECA1, Platelet endothelial cell adhesion molecule | D..YTEV | 1E-08 | 1E-04 | 687 | 67 | 117 | ITIM motif, [VILS].Y..[LVIS], [38],  LIG_SH2, Y[ET][DEQ][IMV], [39] |
| Q13444 | ADA15, Disintegrin and metalloproteinase domain-containing protein 15 | S..P.PP.R | 1E-08 | 2E-04 | 743 | 26 | 96 | LIG_SH3 Class II, P....P..P, [40] |
| Q13443 | ADAM9, Disintegrin and metalloproteinase domain-containing protein 9 | P..P..P.PK | 2E-08 | 2E-04 | 787 | 69 | 100 | LIG_SH3, PPP[AILVP]PPPP[AILVP]P, [41] |
| O43184 | ADA12, Disintegrin and metalloproteinase domain-containing protein 12 | DIS.P | 5E-08 | 3E-04 | 799 | 70 | 179 | LIG_14-3-3, R.[SYFWTQAD].[ST].[PLM], [42] |
| P25189 | MYP0, Myelin protein P0 | KK.KG.G | 8E-08 | 3E-04 | 235 | 56 | 68 | ER export motif of Type II membrane proteins, [RK].[RK]....$, [43] |
| O60500 | NPHN, Nephrin | D..P.L.P..E | 1E-08 | 4E-04 | 1144 | 68 | 164 | LIG_MYND, P.L.P, [44] |
| Q96RL6 | SIG11, Sialic acid-binding Ig-like lectin 11 | EL.YA.L | 2E-08 | 4E-04 | 629 | 57 | 113 | LIG_SH2, L.YA.L , [45,46] |
| Q08554 | DSC1, Desmocollin-1 | NTE.PG | 2E-08 | 5E-04 | 737 | 23 | 179 |  |
| Q02487 | DSC2, Desmocollin-2 | TEAPG | 4E-08 | 5E-04 | 742 | 27 | 185 |  |
| Q6EMK4 | VASN, Vasorin | P.E..G.K..L | 4E-08 | 6E-04 | 621 | 25 | 76 |  |
| Q9UIQ6 | LCAP, Leucyl-cystinyl aminopeptidase | S.MNRS | 7E-08 | 6E-04 | 80 | 25 | 109 |  |
| Q9UN42 | AT1B4, X/potassium-transporting ATPase subunit beta-m | E..A.EEA | 9E-08 | 7E-04 | 36 | 67 | 109 | LIG_TRAF2, [PSAT].[QE]E, [47] |
| O75096 | LRP4, Low-density lipoprotein receptor-related protein 4 | LT..NP.Y | 5E-08 | 8E-04 | 1807 | 16 | 158 | LIG_PTB_1, NP.Y, [48] |
