# Supplementary material for: Protein Disorder and Short Conserved Motifs in Disordered Regions Are Enriched near the Cytoplasmic Side of Single-Pass Transmembrane Proteins
Source: PLoS One. 2012 Sep 4;7(9):e44389. doi: 10.1371/journal.pone.0044389 (PMC3433447; doi:10.1371/journal.pone.0044389)
Supplement: Table S2 — Protein dataset used in the present study. (DOC) [file pone.0044389.s005.doc]

| **Uniprot ID** | **Name** | **Protein name** | |
| --- | --- | --- | --- |
| P56937 | DHB7_HUMAN | 3-keto-steroid reductase | |
| P08195 | 4F2_HUMAN | 4F2 cell-surface antigen heavy chain | |
| Q04771 | ACVR1_HUMAN | Activin receptor type-1 | |
| P36896 | ACV1B_HUMAN | Activin receptor type-1B | |
| Q8NER5 | ACV1C_HUMAN | Activin receptor type-1C | |
| P27037 | AVR2A_HUMAN | Activin receptor type-2A | |
| Q13705 | AVR2B_HUMAN | Activin receptor type-2B | |
| Q9H6B4 | ACAM_HUMAN | Adipocyte adhesion molecule | |
| Q9HDC9 | APMAP_HUMAN | Adipocyte plasma membrane-associated protein | |
| P28907 | CD38_HUMAN | ADP-ribosyl cyclase 1 | |
| Q15109 | RAGE_HUMAN | Advanced glycosylation end product-specific receptor | |
| Q16586 | SGCA_HUMAN | Alpha-sarcoglycan | |
| P16234 | PGFRA_HUMAN | Alpha-type platelet-derived growth factor receptor | |
| Q86WK6 | AMGO1_HUMAN | Amphoterin-induced protein 1 | |
| P05067 | A4_HUMAN | Amyloid beta A4 protein | |
| P51693 | APLP1_HUMAN | Amyloid-like protein 1 | |
| Q06481 | APLP2_HUMAN | Amyloid-like protein 2 | |
| Q02763 | TIE2_HUMAN | Angiopoietin-1 receptor | |
| P12821 | ACE_HUMAN | Angiotensin-converting enzyme | |
| Q9BYF1 | ACE2_HUMAN | Angiotensin-converting enzyme 2 | |
| P58335 | ANTR2_HUMAN | Anthrax toxin receptor 2 | |
| Q6UW56 | APR3_HUMAN | Apoptosis-related protein 3 | |
| P07306 | ASGR1_HUMAN | Asialoglycoprotein receptor 1 | |
| P16066 | ANPRA_HUMAN | Atrial natriuretic peptide receptor 1 | |
| P20594 | ANPRB_HUMAN | Atrial natriuretic peptide receptor 2 | |
| P17342 | ANPRC_HUMAN | Atrial natriuretic peptide receptor 3 | |
| Q9Y5Q5 | CORIN_HUMAN | Atrial natriuretic peptide-converting enzyme | |
| O75882 | ATRN_HUMAN | Attractin |  |
| Q5VV63 | ATRN1_HUMAN | Attractin-like protein 1 | |
| P15391 | CD19_HUMAN | B-lymphocyte antigen CD19 | |
| P11362 | FGFR1_HUMAN | Basic fibroblast growth factor receptor 1 | |
| P35613 | BASI_HUMAN | Basigin |  |
| Q16620 | NTRK2_HUMAN | BDNF/NT-3 growth factors receptor | |
| Q16585 | SGCB_HUMAN | Beta-sarcoglycan | |
| P56817 | BACE1_HUMAN | Beta-secretase 1 | |
| Q9Y5Z0 | BACE2_HUMAN | Beta-secretase 2 | |
| P09619 | PGFRB_HUMAN | Beta-type platelet-derived growth factor receptor | |
| Q13145 | BAMBI_HUMAN | BMP and activin membrane-bound inhibitor homolog | |
| P36894 | BMR1A_HUMAN | Bone morphogenetic protein receptor type-1A | |
| O00238 | BMR1B_HUMAN | Bone morphogenetic protein receptor type-1B | |
| Q13873 | BMPR2_HUMAN | Bone morphogenetic protein receptor type-2 | |
| Q9BWV1 | BOC_HUMAN | Brother of CDO | |
| Q13410 | BT1A1_HUMAN | Butyrophilin subfamily 1 member A1 | |
| Q7KYR7 | BT2A1_HUMAN | Butyrophilin subfamily 2 member A1 | |
| O00481 | BT3A1_HUMAN | Butyrophilin subfamily 3 member A1 | |
| P78410 | BT3A2_HUMAN | Butyrophilin subfamily 3 member A2 | |
| O00478 | BT3A3_HUMAN | Butyrophilin subfamily 3 member A3 | |
| Q9UIR0 | BTNL2_HUMAN | Butyrophilin-like protein 2 | |
| Q6UXG8 | BTNL9_HUMAN | Butyrophilin-like protein 9 | |
| Q5QGZ9 | CL12A_HUMAN | C-type lectin domain family 12 member A | |
| Q2HXU8 | CL12B_HUMAN | C-type lectin domain family 12 member B | |
| Q9UHP7 | CLC2D_HUMAN | C-type lectin domain family 2 member D | |
| Q9H2X3 | CLC4M_HUMAN | C-type lectin domain family 4 member M | |
| Q6EIG7 | CLC6A_HUMAN | C-type lectin domain family 6 member A | |
| Q9BXN2 | CLC7A_HUMAN | C-type lectin domain family 7 member A | |
| Q9UBG0 | MRC2_HUMAN | C-type mannose receptor 2 | |
| P12830 | CADH1_HUMAN | Cadherin-1 | |
| Q9Y6N8 | CAD10_HUMAN | Cadherin-10 | |
| P55287 | CAD11_HUMAN | Cadherin-11 | |
| P55289 | CAD12_HUMAN | Cadherin-12 | |
| P55291 | CAD15_HUMAN | Cadherin-15 | |
| O75309 | CAD16_HUMAN | Cadherin-16 | |
| Q13634 | CAD18_HUMAN | Cadherin-18 | |
| Q9H159 | CAD19_HUMAN | Cadherin-19 | |
| P19022 | CADH2_HUMAN | Cadherin-2 | |
| Q9HBT6 | CAD20_HUMAN | Cadherin-20 | |
| Q9UJ99 | CAD22_HUMAN | Cadherin-22 | |
| Q9H251 | CAD23_HUMAN | Cadherin-23 | |
| P55283 | CADH4_HUMAN | Cadherin-4 | |
| P33151 | CADH5_HUMAN | Cadherin-5 | |
| P55285 | CADH6_HUMAN | Cadherin-6 | |
| Q9ULB5 | CADH7_HUMAN | Cadherin-7 | |
| P55286 | CADH8_HUMAN | Cadherin-8 | |
| O94985 | CSTN1_HUMAN | Calsyntenin-1 | |
| Q9H4D0 | CSTN2_HUMAN | Calsyntenin-2 | |
| Q9BQT9 | CSTN3_HUMAN | Calsyntenin-3 | |
| O43570 | CAH12_HUMAN | Carbonic anhydrase 12 | |
| Q9ULX7 | CAH14_HUMAN | Carbonic anhydrase 14 | |
| O75976 | CBPD_HUMAN | Carboxypeptidase D | |
| P13688 | CEAM1_HUMAN | Carcinoembryonic antigen-related cell adhesion molecule 1 | |
| Q13740 | CD166_HUMAN | CD166 antigen | |
| Q9NNX6 | CD209_HUMAN | CD209 antigen | |
| Q8IX05 | CD302_HUMAN | CD302 antigen | |
| P29965 | CD40L_HUMAN | CD40 ligand | |
| P16070 | CD44_HUMAN | CD44 antigen | |
| Q01151 | CD83_HUMAN | CD83 antigen | |
| Q8TCZ2 | C99L2_HUMAN | CD99 antigen-like protein 2 | |
| Q9BY67 | CADM1_HUMAN | Cell adhesion molecule 1 | |
| Q8N3J6 | CADM2_HUMAN | Cell adhesion molecule 2 | |
| Q8N126 | CADM3_HUMAN | Cell adhesion molecule 3 | |
| Q4KMG0 | CDON_HUMAN | Cell adhesion molecule-related/down-regulated by oncogenes | |
| A0ZSE6 | CC50C_HUMAN | Cell cycle control protein 50C | |
| Q99795 | GPA33_HUMAN | Cell surface A33 antigen | |
| P43121 | MUC18_HUMAN | Cell surface glycoprotein MUC18 | |
| Q6UVK1 | CSPG4_HUMAN | Chondroitin sulfate proteoglycan 4 | |
| Q9H9P2 | CHODL_HUMAN | Chondrolectin | |
| Q9UMD9 | COHA1_HUMAN | Collagen alpha-1(XVII) chain | |
| Q5KU26 | COL12_HUMAN | Collectin-12 |  |
| Q9HBJ8 | TMM27_HUMAN | Collectrin |  |
| P17927 | CR1_HUMAN | Complement receptor type 1 | |
| P20023 | CR2_HUMAN | Complement receptor type 2 | |
| P78357 | CNTP1_HUMAN | Contactin-associated protein 1 | |
| Q9UHC6 | CNTP2_HUMAN | Contactin-associated protein-like 2 | |
| Q9BZ76 | CNTP3_HUMAN | Contactin-associated protein-like 3 | |
| Q96NU0 | CNT3B_HUMAN | Contactin-associated protein-like 3B | |
| Q9C0A0 | CNTP4_HUMAN | Contactin-associated protein-like 4 | |
| Q8WYK1 | CNTP5_HUMAN | Contactin-associated protein-like 5 | |
| P78310 | CXAR_HUMAN | Coxsackievirus and adenovirus receptor | |
| P82279 | CRUM1_HUMAN | Crumbs homolog 1 | |
| Q5IJ48 | CRUM2_HUMAN | Crumbs homolog 2 | |
| Q96J86 | CYYR1_HUMAN | Cysteine and tyrosine-rich protein 1 | |
| P31785 | IL2RG_HUMAN | Cytokine receptor common subunit gamma | |
| P16410 | CTLA4_HUMAN | Cytotoxic T-lymphocyte protein 4 | |
| O00548 | DLL1_HUMAN | Delta-like protein 1 | |
| Q9NR61 | DLL4_HUMAN | Delta-like protein 4 | |
| Q92629 | SGCD_HUMAN | Delta-sarcoglycan | |
| Q08554 | DSC1_HUMAN | Desmocollin-1 | |
| Q02487 | DSC2_HUMAN | Desmocollin-2 | |
| Q14574 | DSC3_HUMAN | Desmocollin-3 | |
| P42658 | DPP6_HUMAN | Dipeptidyl aminopeptidase-like protein 6 | |
| P27487 | DPP4_HUMAN | Dipeptidyl peptidase 4 | |
| Q16832 | DDR2_HUMAN | Discoidin domain-containing receptor 2 | |
| Q96PD2 | DCBD2_HUMAN | Discoidin, CUB and LCCL domain-containing protein 2 | |
| O14672 | ADA10_HUMAN | Disintegrin and metalloproteinase domain-containing protein 10 | |
| O75078 | ADA11_HUMAN | Disintegrin and metalloproteinase domain-containing protein 11 | |
| O43184 | ADA12_HUMAN | Disintegrin and metalloproteinase domain-containing protein 12 | |
| Q13444 | ADA15_HUMAN | Disintegrin and metalloproteinase domain-containing protein 15 | |
| P78536 | ADA17_HUMAN | Disintegrin and metalloproteinase domain-containing protein 17 | |
| Q9Y3Q7 | ADA18_HUMAN | Disintegrin and metalloproteinase domain-containing protein 18 | |
| Q9H013 | ADA19_HUMAN | Disintegrin and metalloproteinase domain-containing protein 19 | |
| Q99965 | ADAM2_HUMAN | Disintegrin and metalloproteinase domain-containing protein 2 | |
| O43506 | ADA20_HUMAN | Disintegrin and metalloproteinase domain-containing protein 20 | |
| Q9UKJ8 | ADA21_HUMAN | Disintegrin and metalloproteinase domain-containing protein 21 | |
| O75077 | ADA23_HUMAN | Disintegrin and metalloproteinase domain-containing protein 23 | |
| Q9UKQ2 | ADA28_HUMAN | Disintegrin and metalloproteinase domain-containing protein 28 | |
| Q9UKF5 | ADA29_HUMAN | Disintegrin and metalloproteinase domain-containing protein 29 | |
| Q9UKF2 | ADA30_HUMAN | Disintegrin and metalloproteinase domain-containing protein 30 | |
| Q8TC27 | ADA32_HUMAN | Disintegrin and metalloproteinase domain-containing protein 32 | |
| Q9BZ11 | ADA33_HUMAN | Disintegrin and metalloproteinase domain-containing protein 33 | |
| Q9H2U9 | ADAM7_HUMAN | Disintegrin and metalloproteinase domain-containing protein 7 | |
| P78325 | ADAM8_HUMAN | Disintegrin and metalloproteinase domain-containing protein 8 | |
| Q13443 | ADAM9_HUMAN | Disintegrin and metalloproteinase domain-containing protein 9 | |
| Q9Y2G8 | DJC16_HUMAN | DnaJ homolog subfamily C member 16 | |
| O60469 | DSCAM_HUMAN | Down syndrome cell adhesion molecule | |
| Q8TD84 | DSCL1_HUMAN | Down syndrome cell adhesion molecule-like protein 1 | |
| O75923 | DYSF_HUMAN | Dysferlin | |
| Q14118 | DAG1_HUMAN | Dystroglycan | |
| P16581 | LYAM2_HUMAN | E-selectin | |
| Q07108 | CD69_HUMAN | Early activation antigen CD69 | |
| P22413 | ENPP1_HUMAN | Ectonucleotide pyrophosphatase/phosphodiesterase family member 1 | |
| O14638 | ENPP3_HUMAN | Ectonucleotide pyrophosphatase/phosphodiesterase family member 3 | |
| Q9Y6X5 | ENPP4_HUMAN | Ectonucleotide pyrophosphatase/phosphodiesterase family member 4 | |
| Q6UWR7 | ENPP6_HUMAN | Ectonucleotide pyrophosphatase/phosphodiesterase family member 6 | |
| P17813 | EGLN_HUMAN | Endoglin | |
| Q96AP7 | ESAM_HUMAN | Endothelial cell-selective adhesion molecule | |
| Q9UNN8 | EPCR_HUMAN | Endothelial protein C receptor | |
| P42892 | ECE1_HUMAN | Endothelin-converting enzyme 1 | |
| P98073 | ENTK_HUMAN | Enteropeptidase | |
| P21709 | EPHA1_HUMAN | Ephrin type-A receptor 1 | |
| Q5JZY3 | EPHAA_HUMAN | Ephrin type-A receptor 10 | |
| P29317 | EPHA2_HUMAN | Ephrin type-A receptor 2 | |
| P29320 | EPHA3_HUMAN | Ephrin type-A receptor 3 | |
| P54764 | EPHA4_HUMAN | Ephrin type-A receptor 4 | |
| P54756 | EPHA5_HUMAN | Ephrin type-A receptor 5 | |
| Q9UF33 | EPHA6_HUMAN | Ephrin type-A receptor 6 | |
| Q15375 | EPHA7_HUMAN | Ephrin type-A receptor 7 | |
| P29322 | EPHA8_HUMAN | Ephrin type-A receptor 8 | |
| P54762 | EPHB1_HUMAN | Ephrin type-B receptor 1 | |
| P29323 | EPHB2_HUMAN | Ephrin type-B receptor 2 | |
| P54753 | EPHB3_HUMAN | Ephrin type-B receptor 3 | |
| P54760 | EPHB4_HUMAN | Ephrin type-B receptor 4 | |
| O15197 | EPHB6_HUMAN | Ephrin type-B receptor 6 | |
| P98172 | EFNB1_HUMAN | Ephrin-B1 | |
| P52799 | EFNB2_HUMAN | Ephrin-B2 | |
| Q15768 | EFNB3_HUMAN | Ephrin-B3 | |
| P00533 | EGFR_HUMAN | Epidermal growth factor receptor | |
| P16422 | EPCAM_HUMAN | Epithelial cell adhesion molecule | |
| Q08345 | DDR1_HUMAN | Epithelial discoidin domain-containing receptor 1 | |
| O43556 | SGCE_HUMAN | Epsilon-sarcoglycan | |
| P19235 | EPOR_HUMAN | Erythropoietin receptor | |
| P21802 | FGFR2_HUMAN | Fibroblast growth factor receptor 2 | |
| P22607 | FGFR3_HUMAN | Fibroblast growth factor receptor 3 | |
| P22455 | FGFR4_HUMAN | Fibroblast growth factor receptor 4 | |
| Q8N441 | FGRL1_HUMAN | Fibroblast growth factor receptor-like 1 | |
| P78423 | X3CL1_HUMAN | Fractalkine | |
| Q14802 | FXYD3_HUMAN | FXYD domain-containing ion transport regulator 3 | |
| Q9H0Q3 | FXYD6_HUMAN | FXYD domain-containing ion transport regulator 6 | |
| P36269 | GGT5_HUMAN | Gamma-glutamyltransferase 5 | |
| P19440 | GGT1_HUMAN | Gamma-glutamyltranspeptidase 1 | |
| P36268 | GGT2_HUMAN | Gamma-glutamyltranspeptidase 2 | |
| Q13326 | SGCG_HUMAN | Gamma-sarcoglycan | |
| Q04609 | FOLH1_HUMAN | Glutamate carboxypeptidase 2 | |
| Q07075 | AMPE_HUMAN | Glutamyl aminopeptidase | |
| P02724 | GLPA_HUMAN | Glycophorin-A | |
| Q92896 | GSLG1_HUMAN | Golgi apparatus protein 1 | |
| Q86XS8 | GOLI_HUMAN | Goliath homolog | |
| P10912 | GHR_HUMAN | Growth hormone receptor | |
| P25092 | GUC2C_HUMAN | Heat-stable enterotoxin receptor | |
| Q9UBK5 | HCST_HUMAN | Hematopoietic cell signal transducer | |
| P28906 | CD34_HUMAN | Hematopoietic progenitor cell antigen CD34 | |
| A8MVW5 | HECA2_HUMAN | HEPACAM family member 2 | |
| P08581 | MET_HUMAN | Hepatocyte growth factor receptor | |
| Q30201 | HFE_HUMAN | Hereditary hemochromatosis protein | |
| P60508 | EFR1_HUMAN | HERV-FRD_6p24.1 provirus ancestral Env polyprotein | |
| Q9N2J8 | ENH3_HUMAN | HERV-H_2q24.1 provirus ancestral Env polyprotein | |
| Q9N2K0 | ENH1_HUMAN | HERV-H_2q24.3 provirus ancestral Env polyprotein | |
| Q9N2J9 | ENH2_HUMAN | HERV-H_3q26 provirus ancestral Env polyprotein | |
| P61550 | ENT1_HUMAN | HERV-T_19q13.11 provirus ancestral Env polyprotein | |
| Q9UQF0 | ENW1_HUMAN | HERV-W_7q21.2 provirus ancestral Env polyprotein | |
| P12319 | FCERA_HUMAN | High affinity immunoglobulin epsilon receptor subunit alpha | |
| P30273 | FCERG_HUMAN | High affinity immunoglobulin epsilon receptor subunit gamma | |
| P12314 | FCGR1_HUMAN | High affinity immunoglobulin gamma Fc receptor I | |
| P04629 | NTRK1_HUMAN | High affinity nerve growth factor receptor | |
| P30443 | 1A01_HUMAN | HLA class I histocompatibility antigen, A-1 alpha chain | |
| P13746 | 1A11_HUMAN | HLA class I histocompatibility antigen, A-11 alpha chain | |
| P01892 | 1A02_HUMAN | HLA class I histocompatibility antigen, A-2 alpha chain | |
| P30447 | 1A23_HUMAN | HLA class I histocompatibility antigen, A-23 alpha chain | |
| P05534 | 1A24_HUMAN | HLA class I histocompatibility antigen, A-24 alpha chain | |
| P18462 | 1A25_HUMAN | HLA class I histocompatibility antigen, A-25 alpha chain | |
| P30450 | 1A26_HUMAN | HLA class I histocompatibility antigen, A-26 alpha chain | |
| P30512 | 1A29_HUMAN | HLA class I histocompatibility antigen, A-29 alpha chain | |
| P04439 | 1A03_HUMAN | HLA class I histocompatibility antigen, A-3 alpha chain | |
| P16188 | 1A30_HUMAN | HLA class I histocompatibility antigen, A-30 alpha chain | |
| P16189 | 1A31_HUMAN | HLA class I histocompatibility antigen, A-31 alpha chain | |
| P10314 | 1A32_HUMAN | HLA class I histocompatibility antigen, A-32 alpha chain | |
| P16190 | 1A33_HUMAN | HLA class I histocompatibility antigen, A-33 alpha chain | |
| P30453 | 1A34_HUMAN | HLA class I histocompatibility antigen, A-34 alpha chain | |
| P30455 | 1A36_HUMAN | HLA class I histocompatibility antigen, A-36 alpha chain | |
| P30456 | 1A43_HUMAN | HLA class I histocompatibility antigen, A-43 alpha chain | |
| P30457 | 1A66_HUMAN | HLA class I histocompatibility antigen, A-66 alpha chain | |
| P01891 | 1A68_HUMAN | HLA class I histocompatibility antigen, A-68 alpha chain | |
| P10316 | 1A69_HUMAN | HLA class I histocompatibility antigen, A-69 alpha chain | |
| P30459 | 1A74_HUMAN | HLA class I histocompatibility antigen, A-74 alpha chain | |
| Q09160 | 1A80_HUMAN | HLA class I histocompatibility antigen, A-80 alpha chain | |
| P13747 | HLAE_HUMAN | HLA class I histocompatibility antigen, alpha chain E | |
| P30511 | HLAF_HUMAN | HLA class I histocompatibility antigen, alpha chain F | |
| P30461 | 1B13_HUMAN | HLA class I histocompatibility antigen, B-13 alpha chain | |
| P30462 | 1B14_HUMAN | HLA class I histocompatibility antigen, B-14 alpha chain | |
| P30464 | 1B15_HUMAN | HLA class I histocompatibility antigen, B-15 alpha chain | |
| P30466 | 1B18_HUMAN | HLA class I histocompatibility antigen, B-18 alpha chain | |
| P03989 | 1B27_HUMAN | HLA class I histocompatibility antigen, B-27 alpha chain | |
| P30685 | 1B35_HUMAN | HLA class I histocompatibility antigen, B-35 alpha chain | |
| P18463 | 1B37_HUMAN | HLA class I histocompatibility antigen, B-37 alpha chain | |
| Q95365 | 1B38_HUMAN | HLA class I histocompatibility antigen, B-38 alpha chain | |
| P30475 | 1B39_HUMAN | HLA class I histocompatibility antigen, B-39 alpha chain | |
| Q04826 | 1B40_HUMAN | HLA class I histocompatibility antigen, B-40 alpha chain | |
| P30479 | 1B41_HUMAN | HLA class I histocompatibility antigen, B-41 alpha chain | |
| P30480 | 1B42_HUMAN | HLA class I histocompatibility antigen, B-42 alpha chain | |
| P30483 | 1B45_HUMAN | HLA class I histocompatibility antigen, B-45 alpha chain | |
| P30484 | 1B46_HUMAN | HLA class I histocompatibility antigen, B-46 alpha chain | |
| P30485 | 1B47_HUMAN | HLA class I histocompatibility antigen, B-47 alpha chain | |
| P30486 | 1B48_HUMAN | HLA class I histocompatibility antigen, B-48 alpha chain | |
| P30487 | 1B49_HUMAN | HLA class I histocompatibility antigen, B-49 alpha chain | |
| P30488 | 1B50_HUMAN | HLA class I histocompatibility antigen, B-50 alpha chain | |
| P18464 | 1B51_HUMAN | HLA class I histocompatibility antigen, B-51 alpha chain | |
| P30490 | 1B52_HUMAN | HLA class I histocompatibility antigen, B-52 alpha chain | |
| P30491 | 1B53_HUMAN | HLA class I histocompatibility antigen, B-53 alpha chain | |
| P30492 | 1B54_HUMAN | HLA class I histocompatibility antigen, B-54 alpha chain | |
| P30493 | 1B55_HUMAN | HLA class I histocompatibility antigen, B-55 alpha chain | |
| P30495 | 1B56_HUMAN | HLA class I histocompatibility antigen, B-56 alpha chain | |
| P18465 | 1B57_HUMAN | HLA class I histocompatibility antigen, B-57 alpha chain | |
| P10319 | 1B58_HUMAN | HLA class I histocompatibility antigen, B-58 alpha chain | |
| Q29940 | 1B59_HUMAN | HLA class I histocompatibility antigen, B-59 alpha chain | |
| Q29836 | 1B67_HUMAN | HLA class I histocompatibility antigen, B-67 alpha chain | |
| P01889 | 1B07_HUMAN | HLA class I histocompatibility antigen, B-7 alpha chain | |
| P30498 | 1B78_HUMAN | HLA class I histocompatibility antigen, B-78 alpha chain | |
| P30460 | 1B08_HUMAN | HLA class I histocompatibility antigen, B-8 alpha chain | |
| Q31610 | 1B81_HUMAN | HLA class I histocompatibility antigen, B-81 alpha chain | |
| Q29718 | 1B82_HUMAN | HLA class I histocompatibility antigen, B-82 alpha chain | |
| P30499 | 1C01_HUMAN | HLA class I histocompatibility antigen, Cw-1 alpha chain | |
| P30508 | 1C12_HUMAN | HLA class I histocompatibility antigen, Cw-12 alpha chain | |
| P30510 | 1C14_HUMAN | HLA class I histocompatibility antigen, Cw-14 alpha chain | |
| Q07000 | 1C15_HUMAN | HLA class I histocompatibility antigen, Cw-15 alpha chain | |
| Q29960 | 1C16_HUMAN | HLA class I histocompatibility antigen, Cw-16 alpha chain | |
| Q95604 | 1C17_HUMAN | HLA class I histocompatibility antigen, Cw-17 alpha chain | |
| Q29865 | 1C18_HUMAN | HLA class I histocompatibility antigen, Cw-18 alpha chain | |
| P30501 | 1C02_HUMAN | HLA class I histocompatibility antigen, Cw-2 alpha chain | |
| P04222 | 1C03_HUMAN | HLA class I histocompatibility antigen, Cw-3 alpha chain | |
| P30504 | 1C04_HUMAN | HLA class I histocompatibility antigen, Cw-4 alpha chain | |
| Q9TNN7 | 1C05_HUMAN | HLA class I histocompatibility antigen, Cw-5 alpha chain | |
| Q29963 | 1C06_HUMAN | HLA class I histocompatibility antigen, Cw-6 alpha chain | |
| P10321 | 1C07_HUMAN | HLA class I histocompatibility antigen, Cw-7 alpha chain | |
| P30505 | 1C08_HUMAN | HLA class I histocompatibility antigen, Cw-8 alpha chain | |
| P04233 | HG2A_HUMAN | HLA class II histocompatibility antigen gamma chain | |
| P06340 | 2DOA_HUMAN | HLA class II histocompatibility antigen, DO alpha chain | |
| P13765 | 2DOB_HUMAN | HLA class II histocompatibility antigen, DO beta chain | |
| P20036 | DPA1_HUMAN | HLA class II histocompatibility antigen, DP alpha 1 chain | |
| P04440 | DPB1_HUMAN | HLA class II histocompatibility antigen, DP beta 1 chain | |
| P01909 | 2DA1_HUMAN | HLA class II histocompatibility antigen, DQ alpha 1 chain | |
| P01906 | 2DA2_HUMAN | HLA class II histocompatibility antigen, DQ alpha 2 chain | |
| P01920 | DQB1_HUMAN | HLA class II histocompatibility antigen, DQ beta 1 chain | |
| P05538 | DQB2_HUMAN | HLA class II histocompatibility antigen, DQ beta 2 chain | |
| P01908 | HA21_HUMAN | HLA class II histocompatibility antigen, DQ(1) alpha chain | |
| P05536 | HA27_HUMAN | HLA class II histocompatibility antigen, DQ(W3) alpha chain | |
| P01903 | 2DRA_HUMAN | HLA class II histocompatibility antigen, DR alpha chain | |
| P79483 | DRB3_HUMAN | HLA class II histocompatibility antigen, DR beta 3 chain | |
| P13762 | DRB4_HUMAN | HLA class II histocompatibility antigen, DR beta 4 chain | |
| Q30154 | DRB5_HUMAN | HLA class II histocompatibility antigen, DR beta 5 chain | |
| P01914 | HB2C_HUMAN | HLA class II histocompatibility antigen, DR-1 beta chain | |
| P04229 | 2B11_HUMAN | HLA class II histocompatibility antigen, DRB1-1 beta chain | |
| Q30167 | 2B1A_HUMAN | HLA class II histocompatibility antigen, DRB1-10 beta chain | |
| P20039 | 2B1B_HUMAN | HLA class II histocompatibility antigen, DRB1-11 beta chain | |
| P01911 | 2B1F_HUMAN | HLA class II histocompatibility antigen, DRB1-15 beta chain | |
| P01912 | HB2B_HUMAN | HLA class II histocompatibility antigen, DRB1-3 chain | |
| P13760 | 2B14_HUMAN | HLA class II histocompatibility antigen, DRB1-4 beta chain | |
| P13761 | 2B17_HUMAN | HLA class II histocompatibility antigen, DRB1-7 beta chain | |
| Q30134 | 2B18_HUMAN | HLA class II histocompatibility antigen, DRB1-8 beta chain | |
| Q9TQE0 | 2B19_HUMAN | HLA class II histocompatibility antigen, DRB1-9 beta chain | |
| P55899 | FCGRN_HUMAN | IgG receptor FcRn large subunit p51 | |
| Q5DX21 | IGS11_HUMAN | Immunoglobulin superfamily member 11 | |
| Q86SU0 | ILDR1_HUMAN | Immunoglobulin-like domain-containing receptor 1 | |
| Q8N608 | DPP10_HUMAN | Inactive dipeptidyl peptidase 10 | |
| Q9Y6W8 | ICOS_HUMAN | Inducible T-cell costimulator | |
| P06213 | INSR_HUMAN | Insulin receptor | |
| P14616 | INSRR_HUMAN | Insulin receptor-related protein | |
| P08069 | IGF1R_HUMAN | Insulin-like growth factor 1 receptor | |
| P98153 | IDD_HUMAN | Integral membrane protein DGCR2/IDD | |
| P17301 | ITA2_HUMAN | Integrin alpha-2 | |
| P26006 | ITA3_HUMAN | Integrin alpha-3 | |
| P13612 | ITA4_HUMAN | Integrin alpha-4 | |
| P08648 | ITA5_HUMAN | Integrin alpha-5 | |
| P23229 | ITA6_HUMAN | Integrin alpha-6 | |
| Q13683 | ITA7_HUMAN | Integrin alpha-7 | |
| P53708 | ITA8_HUMAN | Integrin alpha-8 | |
| Q13797 | ITA9_HUMAN | Integrin alpha-9 | |
| Q13349 | ITAD_HUMAN | Integrin alpha-D | |
| P08514 | ITA2B_HUMAN | Integrin alpha-IIb | |
| P20701 | ITAL_HUMAN | Integrin alpha-L | |
| P11215 | ITAM_HUMAN | Integrin alpha-M | |
| P06756 | ITAV_HUMAN | Integrin alpha-V | |
| P20702 | ITAX_HUMAN | Integrin alpha-X | |
| P05556 | ITB1_HUMAN | Integrin beta-1 | |
| P05107 | ITB2_HUMAN | Integrin beta-2 | |
| P05106 | ITB3_HUMAN | Integrin beta-3 | |
| P18084 | ITB5_HUMAN | Integrin beta-5 | |
| P18564 | ITB6_HUMAN | Integrin beta-6 | |
| P26010 | ITB7_HUMAN | Integrin beta-7 | |
| P05362 | ICAM1_HUMAN | Intercellular adhesion molecule 1 | |
| P13598 | ICAM2_HUMAN | Intercellular adhesion molecule 2 | |
| P32942 | ICAM3_HUMAN | Intercellular adhesion molecule 3 | |
| P17181 | INAR1_HUMAN | Interferon alpha/beta receptor 1 | |
| P48551 | INAR2_HUMAN | Interferon alpha/beta receptor 2 | |
| P15260 | INGR1_HUMAN | Interferon gamma receptor 1 | |
| P38484 | INGR2_HUMAN | Interferon gamma receptor 2 | |
| Q9NPH3 | IL1AP_HUMAN | Interleukin-1 receptor accessory protein | |
| Q9NZN1 | IRPL1_HUMAN | Interleukin-1 receptor accessory protein-like 1 | |
| P14778 | IL1R1_HUMAN | Interleukin-1 receptor type 1 | |
| P27930 | IL1R2_HUMAN | Interleukin-1 receptor type 2 | |
| Q9HB29 | ILRL2_HUMAN | Interleukin-1 receptor-like 2 | |
| Q08334 | I10R2_HUMAN | Interleukin-10 receptor subunit beta | |
| Q14626 | I11RA_HUMAN | Interleukin-11 receptor subunit alpha | |
| Q99665 | I12R2_HUMAN | Interleukin-12 receptor subunit beta-2 | |
| P78552 | I13R1_HUMAN | Interleukin-13 receptor subunit alpha-1 | |
| Q14627 | I13R2_HUMAN | Interleukin-13 receptor subunit alpha-2 | |
| Q13478 | IL18R_HUMAN | Interleukin-18 receptor 1 | |
| P01589 | IL2RA_HUMAN | Interleukin-2 receptor subunit alpha | |
| P24394 | IL4RA_HUMAN | Interleukin-4 receptor subunit alpha | |
| Q01344 | IL5RA_HUMAN | Interleukin-5 receptor subunit alpha | |
| P08887 | IL6RA_HUMAN | Interleukin-6 receptor subunit alpha | |
| P40189 | IL6RB_HUMAN | Interleukin-6 receptor subunit beta | |
| P16871 | IL7RA_HUMAN | Interleukin-7 receptor subunit alpha | |
| Q6PHW0 | IYD1_HUMAN | Iodotyrosine dehalogenase 1 | |
| Q9Y624 | JAM1_HUMAN | Junctional adhesion molecule A | |
| P57087 | JAM2_HUMAN | Junctional adhesion molecule B | |
| Q9BX67 | JAM3_HUMAN | Junctional adhesion molecule C | |
| Q8NC54 | KCT2_HUMAN | Keratinocyte-associated transmembrane protein 2 | |
| P26715 | NKG2A_HUMAN | KG2-A/KG2-B type II integral membrane protein | |
| Q8N109 | KI2LA_HUMAN | Killer cell immunoglobulin-like receptor 2DL5A | |
| Q8NHK3 | KI2LB_HUMAN | Killer cell immunoglobulin-like receptor 2DL5B | |
| P43629 | KI3L1_HUMAN | Killer cell immunoglobulin-like receptor 3DL1 | |
| P43630 | KI3L2_HUMAN | Killer cell immunoglobulin-like receptor 3DL2 | |
| Q12918 | KLRB1_HUMAN | Killer cell lectin-like receptor subfamily B member 1 | |
| Q9NZS2 | KLRF1_HUMAN | Killer cell lectin-like receptor subfamily F member 1 | |
| Q96J84 | KIRR1_HUMAN | Kin of IRRE-like protein 1 | |
| Q8IZU9 | KIRR3_HUMAN | Kin of IRRE-like protein 3 | |
| P21583 | SCF_HUMAN | Kit ligand | |
| Q96MU8 | KREM1_HUMAN | Kremen protein 1 | |
| O43291 | SPIT2_HUMAN | Kunitz-type protease inhibitor 2 | |
| P14151 | LYAM1_HUMAN | L-selectin | |
| Q6UWM7 | LCTL_HUMAN | Lactase-like protein | |
| P09848 | LPH_HUMAN | Lactase-phlorizin hydrolase | |
| Q9UJQ1 | CT103_HUMAN | LAMP family protein C20orf103 | |
| P48357 | LEPR_HUMAN | Leptin receptor | |
| Q9ULH4 | LRFN2_HUMAN | Leucine-rich repeat and fibronectin type-III domain-containing protein 2 | |
| Q96FE5 | LIGO1_HUMAN | Leucine-rich repeat and immunoglobulin-like domain-containing nogo receptor-interacting protein 1 | |
| Q6UXK5 | LRRN1_HUMAN | Leucine-rich repeat neuronal protein 1 | |
| Q9H3W5 | LRRN3_HUMAN | Leucine-rich repeat neuronal protein 3 | |
| Q86VH5 | LRRT3_HUMAN | Leucine-rich repeat transmembrane neuronal protein 3 | |
| Q86VH4 | LRRT4_HUMAN | Leucine-rich repeat transmembrane neuronal protein 4 | |
| Q9NZU0 | FLRT3_HUMAN | Leucine-rich repeat transmembrane protein FLRT3 | |
| Q8TF66 | LRC15_HUMAN | Leucine-rich repeat-containing protein 15 | |
| Q14392 | LRC32_HUMAN | Leucine-rich repeat-containing protein 32 | |
| Q86YC3 | LRC33_HUMAN | Leucine-rich repeat-containing protein 33 | |
| Q9HBW1 | LRRC4_HUMAN | Leucine-rich repeat-containing protein 4 | |
| Q96JA1 | LRIG1_HUMAN | Leucine-rich repeats and immunoglobulin-like domains protein 1 | |
| O94898 | LRIG2_HUMAN | Leucine-rich repeats and immunoglobulin-like domains protein 2 | |
| Q9UIQ6 | LCAP_HUMAN | Leucyl-cystinyl aminopeptidase | |
| O75019 | LIRA1_HUMAN | Leukocyte immunoglobulin-like receptor subfamily A member 1 | |
| Q8N149 | LIRA2_HUMAN | Leukocyte immunoglobulin-like receptor subfamily A member 2 | |
| Q8NHL6 | LIRB1_HUMAN | Leukocyte immunoglobulin-like receptor subfamily B member 1 | |
| Q86X29 | LSR_HUMAN | Lipolysis-stimulated lipoprotein receptor | |
| Q6PCB7 | S27A1_HUMAN | Long-chain fatty acid transport protein 1 | |
| P31994 | FCG2B_HUMAN | Low affinity immunoglobulin gamma Fc region receptor II-b | |
| P31995 | FCG2C_HUMAN | Low affinity immunoglobulin gamma Fc region receptor II-c | |
| P08637 | FCG3A_HUMAN | Low affinity immunoglobulin gamma Fc region receptor III-A | |
| P01130 | LDLR_HUMAN | Low-density lipoprotein receptor | |
| Q9Y561 | LRP12_HUMAN | Low-density lipoprotein receptor-related protein 12 | |
| Q9NZR2 | LRP1B_HUMAN | Low-density lipoprotein receptor-related protein 1B | |
| P98164 | LRP2_HUMAN | Low-density lipoprotein receptor-related protein 2 | |
| O75096 | LRP4_HUMAN | Low-density lipoprotein receptor-related protein 4 | |
| O75197 | LRP5_HUMAN | Low-density lipoprotein receptor-related protein 5 | |
| O75581 | LRP6_HUMAN | Low-density lipoprotein receptor-related protein 6 | |
| Q14114 | LRP8_HUMAN | Low-density lipoprotein receptor-related protein 8 | |
| O60449 | LY75_HUMAN | Lymphocyte antigen 75 | |
| Q06643 | TNFC_HUMAN | Lymphotoxin-beta | |
| Q7Z3D4 | LYSM3_HUMAN | LysM and putative peptidoglycan-binding domain-containing protein 3 | |
| Q8WWB7 | NCUG1_HUMAN | Lysosomal protein NCU-G1 | |
| P07333 | CSF1R_HUMAN | Macrophage colony-stimulating factor 1 receptor | |
| P22897 | MRC1_HUMAN | Macrophage mannose receptor 1 | |
| Q5VSK2 | MRC1L_HUMAN | Macrophage mannose receptor 1-like protein 1 | |
| Q2M385 | MPEG1_HUMAN | Macrophage-expressed gene 1 protein | |
| Q04912 | RON_HUMAN | Macrophage-stimulating protein receptor | |
| Q95460 | HMR1_HUMAN | Major histocompatibility complex class I-related gene protein | |
| P10721 | KIT_HUMAN | Mast/stem cell growth factor receptor | |
| P50281 | MMP14_HUMAN | Matrix metalloproteinase-14 | |
| P51511 | MMP15_HUMAN | Matrix metalloproteinase-15 | |
| P51512 | MMP16_HUMAN | Matrix metalloproteinase-16 | |
| O75900 | MMP23_HUMAN | Matrix metalloproteinase-23 | |
| Q9Y5R2 | MMP24_HUMAN | Matrix metalloproteinase-24 | |
| Q9BRK3 | MXRA8_HUMAN | Matrix-remodeling-associated protein 8 | |
| P40967 | PME17_HUMAN | Melanocyte protein Pmel 17 | |
| P15529 | MCP_HUMAN | Membrane cofactor protein | |
| Q16853 | AOC3_HUMAN | Membrane primary amine oxidase | |
| Q16819 | MEP1A_HUMAN | Meprin A subunit alpha | |
| Q16820 | MEP1B_HUMAN | Meprin A subunit beta | |
| Q29983 | MICA_HUMAN | MHC class I polypeptide-related sequence A | |
| Q29980 | MICB_HUMAN | MHC class I polypeptide-related sequence B | |
| P55082 | MFAP3_HUMAN | Microfibril-associated glycoprotein 3 | |
| O75121 | MFA3L_HUMAN | Microfibrillar-associated protein 3-like | |
| P15941 | MUC1_HUMAN | Mucin-1 |  |
| Q13477 | MADCA_HUMAN | Mucosal addressin cell adhesion molecule 1 | |
| Q96KG7 | MEG10_HUMAN | Multiple epidermal growth factor-like domains protein 10 | |
| Q7Z7M0 | MEGF8_HUMAN | Multiple epidermal growth factor-like domains protein 8 | |
| O15146 | MUSK_HUMAN | Muscle, skeletal receptor tyrosine-protein kinase | |
| P25189 | MYP0_HUMAN | Myelin protein P0 | |
| O60487 | MPZL2_HUMAN | Myelin protein zero-like protein 2 | |
| P20916 | MAG_HUMAN | Myelin-associated glycoprotein | |
| Q9NZM1 | MYOF_HUMAN | Myoferlin | |
| Q9Y3Q0 | NALD2_HUMAN | N-acetylated-alpha-linked acidic dipeptidase 2 | |
| Q9UQQ1 | NALDL_HUMAN | N-acetylated-alpha-linked acidic dipeptidase-like protein | |
| O76036 | NCTR1_HUMAN | Natural cytotoxicity triggering receptor 1 | |
| O14931 | NCTR3_HUMAN | Natural cytotoxicity triggering receptor 3 | |
| Q13241 | KLRD1_HUMAN | Natural killer cells antigen CD94 | |
| Q92859 | NEO1_HUMAN | Neogenin | |
| O60500 | NPHN_HUMAN | Nephrin | |
| P08473 | NEP_HUMAN | Neprilysin | |
| P43146 | DCC_HUMAN | Netrin receptor DCC | |
| Q8IZJ1 | UNC5B_HUMAN | Netrin receptor UC5B | |
| Q6ZN44 | UNC5A_HUMAN | Netrin receptor UNC5A | |
| O95185 | UNC5C_HUMAN | Netrin receptor UNC5C | |
| P13591 | NCAM1_HUMAN | Neural cell adhesion molecule 1 | |
| O15394 | NCAM2_HUMAN | Neural cell adhesion molecule 2 | |
| P32004 | L1CAM_HUMAN | Neural cell adhesion molecule L1 | |
| O00533 | CHL1_HUMAN | Neural cell adhesion molecule L1-like protein | |
| Q9ULB1 | NRX1A_HUMAN | Neurexin-1-alpha | |
| Q9P2S2 | NRX2A_HUMAN | Neurexin-2-alpha | |
| Q9Y4C0 | NRX3A_HUMAN | Neurexin-3-alpha | |
| O94856 | NFASC_HUMAN | Neurofascin | |
| P46531 | NOTC1_HUMAN | Neurogenic locus notch homolog protein 1 | |
| Q04721 | NOTC2_HUMAN | Neurogenic locus notch homolog protein 2 | |
| Q9UM47 | NOTC3_HUMAN | Neurogenic locus notch homolog protein 3 | |
| Q8N2Q7 | NLGN1_HUMAN | Neuroligin-1 | |
| Q8NFZ4 | NLGN2_HUMAN | Neuroligin-2 | |
| Q9NZ94 | NLGN3_HUMAN | Neuroligin-3 | |
| Q8N0W4 | NLGNX_HUMAN | Neuroligin-4 | |
| Q8NFZ3 | NLGNY_HUMAN | Neuroligin-4, Y linked | |
| Q92823 | NRCAM_HUMAN | Neuronal cell adhesion molecule | |
| O95502 | NPTXR_HUMAN | Neuronal pentraxin receptor | |
| O14786 | NRP1_HUMAN | Neuropilin-1 | |
| O60462 | NRP2_HUMAN | Neuropilin-2 | |
| Q9Y639 | NPTN_HUMAN | Neuroplastin | |
| Q07837 | SLC31_HUMAN | Neutral and basic amino acid transport protein rBAT | |
| Q92542 | NICA_HUMAN | Nicastrin | |
| P26717 | NKG2C_HUMAN | NKG2-C type II integral membrane protein | |
| P26718 | NKG2D_HUMAN | NKG2-D type II integral membrane protein | |
| Q07444 | NKG2E_HUMAN | NKG2-E type II integral membrane protein | |
| Q15155 | NOMO1_HUMAN | Nodal modulator 1 | |
| P69849 | NOMO3_HUMAN | Nodal modulator 3 | |
| Q96E22 | NGBR_HUMAN | Nogo-B receptor | |
| Q9HCG7 | GBA2_HUMAN | Non-lysosomal glucosylceramidase | |
| Q16288 | NTRK3_HUMAN | NT-3 growth factor receptor | |
| Q86WC4 | OSTM1_HUMAN | Osteopetrosis-associated transmembrane protein 1 | |
| Q9HC10 | OTOF_HUMAN | Otoferlin | |
| P41217 | OX2G_HUMAN | OX-2 membrane glycoprotein | |
| P78380 | OLR1_HUMAN | Oxidized low-density lipoprotein receptor 1 | |
| P16109 | LYAM3_HUMAN | P-selectin | |
| Q14242 | SELPL_HUMAN | P-selectin glycoprotein ligand 1 | |
| P78562 | PHEX_HUMAN | Phosphate-regulating neutral endopeptidase | |
| Q96FE7 | P3IP1_HUMAN | Phosphoinositide-3-kinase-interacting protein 1 | |
| O00168 | PLM_HUMAN | Phospholemman | |
| Q6P1J6 | PLB1_HUMAN | Phospholipase B1, membrane-associated | |
| O15162 | PLS1_HUMAN | Phospholipid scramblase 1 | |
| Q9NRY7 | PLS2_HUMAN | Phospholipid scramblase 2 | |
| Q9NRY6 | PLS3_HUMAN | Phospholipid scramblase 3 | |
| Q9NRQ2 | PLS4_HUMAN | Phospholipid scramblase 4 | |
| P53801 | PTTG_HUMAN | Pituitary tumor-transforming gene 1 protein-interacting protein | |
| Q6UQ28 | PLET1_HUMAN | Placenta-expressed transcript 1 protein | |
| P16284 | PECA1_HUMAN | Platelet endothelial cell adhesion molecule | |
| P40197 | GPV_HUMAN | Platelet glycoprotein V | |
| Q9H7M9 | GI24_HUMAN | Platelet receptor Gi24 | |
| Q8IUK5 | PXDC1_HUMAN | Plexin domain-containing protein 1 | |
| Q6UX71 | PXDC2_HUMAN | Plexin domain-containing protein 2 | |
| Q9UIW2 | PLXA1_HUMAN | Plexin-A1 | |
| O75051 | PLXA2_HUMAN | Plexin-A2 | |
| P51805 | PLXA3_HUMAN | Plexin-A3 | |
| Q9HCM2 | PLXA4_HUMAN | Plexin-A4 | |
| Q86YL7 | PDPN_HUMAN | Podoplanin | |
| P15151 | PVR_HUMAN | Poliovirus receptor | |
| Q15223 | PVRL1_HUMAN | Poliovirus receptor-related protein 1 | |
| Q8NA58 | PNDC1_HUMAN | Poly(A)-specific ribonuclease PARN-like domain-containing protein 1 | |
| P01833 | PIGR_HUMAN | Polymeric immunoglobulin receptor | |
| P51164 | ATP4B_HUMAN | Potassium-transporting ATPase subunit beta | |
| P01133 | EGF_HUMAN | Pro-epidermal growth factor | |
| Q02297 | NRG1_HUMAN | Pro-neuregulin-1, membrane-bound isoform | |
| P35070 | BTC_HUMAN | Probetacellulin | |
| Q9NZQ7 | PD1L1_HUMAN | Programmed cell death 1 ligand 1 | |
| Q9BQ51 | PD1L2_HUMAN | Programmed cell death 1 ligand 2 | |
| Q15116 | PDCD1_HUMAN | Programmed cell death protein 1 | |
| Q99075 | HBEGF_HUMAN | Proheparin-binding EGF-like growth factor | |
| P16471 | PRLR_HUMAN | Prolactin receptor | |
| Q07954 | LRP1_HUMAN | Prolow-density lipoprotein receptor-related protein 1 | |
| Q16549 | PCSK7_HUMAN | Proprotein convertase subtilisin/kexin type 7 | |
| Q9BXJ7 | AMNLS_HUMAN | Protein amnionless | |
| Q8J025 | APCD1_HUMAN | Protein APCDD1 | |
| P80370 | DLK1_HUMAN | Protein delta homolog 1 | |
| P22794 | EVI2A_HUMAN | Protein EVI2A | |
| Q5VUB5 | F1711_HUMAN | Protein FAM171A1 | |
| Q6UWH4 | F198B_HUMAN | Protein FAM198B | |
| Q9H0X4 | ITFG3_HUMAN | Protein ITFG3 | |
| P78504 | JAG1_HUMAN | Protein jagged-1 | |
| Q9Y219 | JAG2_HUMAN | Protein jagged-2 | |
| O76095 | JTB_HUMAN | Protein JTB | |
| Q5SGD2 | PPM1L_HUMAN | Protein phosphatase 1L | |
| Q9UBV2 | SE1L1_HUMAN | Protein sel-1 homolog 1 | |
| Q5TEA6 | SE1L2_HUMAN | Protein sel-1 homolog 2 | |
| Q6UWI4 | SHSA2_HUMAN | Protein shisa-2 homolog | |
| Q96DD7 | SHSA4_HUMAN | Protein shisa-4 | |
| Q7Z5N4 | SDK1_HUMAN | Protein sidekick-1 | |
| Q58EX2 | SDK2_HUMAN | Protein sidekick-2 | |
| P60059 | SC61G_HUMAN | Protein transport protein Sec61 subunit gamma | |
| P07949 | RET_HUMAN | Proto-oncogene tyrosine-protein kinase receptor Ret | |
| P08922 | ROS_HUMAN | Proto-oncogene tyrosine-protein kinase ROS | |
| Q9Y5I3 | PCDA1_HUMAN | Protocadherin alpha-1 | |
| Q9Y5I2 | PCDAA_HUMAN | Protocadherin alpha-10 | |
| Q9Y5I1 | PCDAB_HUMAN | Protocadherin alpha-11 | |
| Q9UN75 | PCDAC_HUMAN | Protocadherin alpha-12 | |
| Q9Y5I0 | PCDAD_HUMAN | Protocadherin alpha-13 | |
| Q9Y5H9 | PCDA2_HUMAN | Protocadherin alpha-2 | |
| Q9Y5H8 | PCDA3_HUMAN | Protocadherin alpha-3 | |
| Q9UN74 | PCDA4_HUMAN | Protocadherin alpha-4 | |
| Q9Y5H7 | PCDA5_HUMAN | Protocadherin alpha-5 | |
| Q9UN73 | PCDA6_HUMAN | Protocadherin alpha-6 | |
| Q9UN72 | PCDA7_HUMAN | Protocadherin alpha-7 | |
| Q9Y5H6 | PCDA8_HUMAN | Protocadherin alpha-8 | |
| Q9Y5H5 | PCDA9_HUMAN | Protocadherin alpha-9 | |
| Q9Y5I4 | PCDC2_HUMAN | Protocadherin alpha-C2 | |
| Q9Y5E5 | PCDB4_HUMAN | Protocadherin beta-4 | |
| Q14517 | FAT1_HUMAN | Protocadherin Fat 1 | |
| Q9NYQ8 | FAT2_HUMAN | Protocadherin Fat 2 | |
| Q8TDW7 | FAT3_HUMAN | Protocadherin Fat 3 | |
| Q6V0I7 | FAT4_HUMAN | Protocadherin Fat 4 | |
| Q9Y5H4 | PCDG1_HUMAN | Protocadherin gamma-A1 | |
| Q9Y5H3 | PCDGA_HUMAN | Protocadherin gamma-A10 | |
| Q9Y5H2 | PCDGB_HUMAN | Protocadherin gamma-A11 | |
| O60330 | PCDGC_HUMAN | Protocadherin gamma-A12 | |
| Q9Y5H1 | PCDG2_HUMAN | Protocadherin gamma-A2 | |
| Q9Y5H0 | PCDG3_HUMAN | Protocadherin gamma-A3 | |
| Q9Y5G9 | PCDG4_HUMAN | Protocadherin gamma-A4 | |
| Q9Y5G8 | PCDG5_HUMAN | Protocadherin gamma-A5 | |
| Q9Y5G7 | PCDG6_HUMAN | Protocadherin gamma-A6 | |
| Q9Y5G6 | PCDG7_HUMAN | Protocadherin gamma-A7 | |
| Q9Y5G5 | PCDG8_HUMAN | Protocadherin gamma-A8 | |
| Q9Y5G4 | PCDG9_HUMAN | Protocadherin gamma-A9 | |
| Q9UN71 | PCDGG_HUMAN | Protocadherin gamma-B4 | |
| Q9Y5F8 | PCDGJ_HUMAN | Protocadherin gamma-B7 | |
| Q9UN70 | PCDGK_HUMAN | Protocadherin gamma-C3 | |
| Q9Y5F6 | PCDGM_HUMAN | Protocadherin gamma-C5 | |
| Q08174 | PCDH1_HUMAN | Protocadherin-1 | |
| Q9P2E7 | PCD10_HUMAN | Protocadherin-10 | |
| Q9BZA7 | PC11X_HUMAN | Protocadherin-11 X-linked | |
| Q96QU1 | PCD15_HUMAN | Protocadherin-15 | |
| Q96JQ0 | PCD16_HUMAN | Protocadherin-16 | |
| Q9HCL0 | PCD18_HUMAN | Protocadherin-18 | |
| Q8TAB3 | PCD19_HUMAN | Protocadherin-19 | |
| Q96JP9 | PCD21_HUMAN | Protocadherin-21 | |
| O60245 | PCDH7_HUMAN | Protocadherin-7 | |
| O95206 | PCDH8_HUMAN | Protocadherin-8 | |
| P01135 | TGFA_HUMAN | Protransforming growth factor alpha | |
| A6NGU5 | GGT3_HUMAN | Putative gamma-glutamyltranspeptidase 3 | |
| P01893 | HLAH_HUMAN | Putative HLA class I histocompatibility antigen, alpha chain H | |
| P0C7V7 | SC11B_HUMAN | Putative signal peptidase complex catalytic subunit SEC11B | |
| A6NJW9 | CD8BL_HUMAN | Putative T-cell surface glycoprotein CD8 beta-2 chain | |
| P46695 | IEX1_HUMAN | Radiation-inducible immediate-early gene IEX-1 | |
| O60894 | RAMP1_HUMAN | Receptor activity-modifying protein 1 | |
| O60895 | RAMP2_HUMAN | Receptor activity-modifying protein 2 | |
| O60896 | RAMP3_HUMAN | Receptor activity-modifying protein 3 | |
| P04626 | ERBB2_HUMAN | Receptor tyrosine-protein kinase erbB-2 | |
| P21860 | ERBB3_HUMAN | Receptor tyrosine-protein kinase erbB-3 | |
| Q15303 | ERBB4_HUMAN | Receptor tyrosine-protein kinase erbB-4 | |
| O00559 | RCAS1_HUMAN | Receptor-binding cancer antigen expressed on SiSo cells | |
| P18433 | PTPRA_HUMAN | Receptor-type tyrosine-protein phosphatase alpha | |
| P23467 | PTPRB_HUMAN | Receptor-type tyrosine-protein phosphatase beta | |
| P08575 | PTPRC_HUMAN | Receptor-type tyrosine-protein phosphatase C | |
| P23468 | PTPRD_HUMAN | Receptor-type tyrosine-protein phosphatase delta | |
| P23469 | PTPRE_HUMAN | Receptor-type tyrosine-protein phosphatase epsilon | |
| P10586 | PTPRF_HUMAN | Receptor-type tyrosine-protein phosphatase F | |
| P23470 | PTPRG_HUMAN | Receptor-type tyrosine-protein phosphatase gamma | |
| Q15262 | PTPRK_HUMAN | Receptor-type tyrosine-protein phosphatase kappa | |
| P28827 | PTPRM_HUMAN | Receptor-type tyrosine-protein phosphatase mu | |
| Q16827 | PTPRO_HUMAN | Receptor-type tyrosine-protein phosphatase O | |
| Q15256 | PTPRR_HUMAN | Receptor-type tyrosine-protein phosphatase R | |
| Q13332 | PTPRS_HUMAN | Receptor-type tyrosine-protein phosphatase S | |
| Q16849 | PTPRN_HUMAN | Receptor-type tyrosine-protein phosphatase-like N | |
| Q6ZS82 | R9BP_HUMAN | Regulator of G-protein signaling 9-binding protein | |
| Q8IUW5 | RELL1_HUMAN | RELT-like protein 1 | |
| O75787 | RENR_HUMAN | Renin receptor | |
| Q02846 | GUC2D_HUMAN | Retinal guanylyl cyclase 1 | |
| P51841 | GUC2F_HUMAN | Retinal guanylyl cyclase 2 | |
| P49788 | TIG1_HUMAN | Retinoic acid receptor responder protein 1 | |
| Q9ULK6 | RN150_HUMAN | RING finger protein 150 | |
| Q9Y6N7 | ROBO1_HUMAN | Roundabout homolog 1 | |
| Q14BN4 | SLMAP_HUMAN | Sarcolemmal membrane-associated protein | |
| Q86VB7 | C163A_HUMAN | Scavenger receptor cysteine-rich type 1 protein M130 | |
| Q13018 | PLA2R_HUMAN | Secretory phospholipase A2 receptor | |
| Q9H3S1 | SEM4A_HUMAN | Semaphorin-4A | |
| Q92854 | SEM4D_HUMAN | Semaphorin-4D | |
| Q13591 | SEM5A_HUMAN | Semaphorin-5A | |
| Q9P283 | SEM5B_HUMAN | Semaphorin-5B | |
| Q8NFY4 | SEM6D_HUMAN | Semaphorin-6D | |
| Q12884 | SEPR_HUMAN | Seprase | |
| P05981 | HEPS_HUMAN | Serine protease hepsin | |
| P37023 | ACVL1_HUMAN | Serine/threonine-protein kinase receptor R3 | |
| Q96LC7 | SIG10_HUMAN | Sialic acid-binding Ig-like lectin 10 | |
| Q96RL6 | SIG11_HUMAN | Sialic acid-binding Ig-like lectin 11 | |
| Q13291 | SLAF1_HUMAN | Signaling lymphocytic activation molecule | |
| Q6IA17 | SIGIR_HUMAN | Single Ig IL-1-related receptor | |
| P49771 | FLT3L_HUMAN | SL cytokine | |
| Q0VAQ4 | SMAGP_HUMAN | Small cell adhesion glycoprotein | |
| O60939 | SCN2B_HUMAN | Sodium channel subunit beta-2 | |
| Q9NY72 | SCN3B_HUMAN | Sodium channel subunit beta-3 | |
| Q8IWT1 | SCN4B_HUMAN | Sodium channel subunit beta-4 | |
| P05026 | AT1B1_HUMAN | Sodium/potassium-transporting ATPase subunit beta-1 | |
| P14415 | AT1B2_HUMAN | Sodium/potassium-transporting ATPase subunit beta-2 | |
| P54709 | AT1B3_HUMAN | Sodium/potassium-transporting ATPase subunit beta-3 | |
| Q99523 | SORT_HUMAN | Sortilin | |
| Q92673 | SORL_HUMAN | Sortilin-related receptor | |
| Q8IXA5 | SACA3_HUMAN | Sperm acrosome membrane-associated protein 3 | |
| Q9Y6X1 | SERP1_HUMAN | Stress-associated endoplasmic reticulum protein 1 | |
| Q13586 | STIM1_HUMAN | Stromal interaction molecule 1 | |
| Q9Y5Y6 | ST14_HUMAN | Suppressor of tumorigenicity 14 protein | |
| Q9UGT4 | SUSD2_HUMAN | Sushi domain-containing protein 2 | |
| Q5VX71 | SUSD4_HUMAN | Sushi domain-containing protein 4 | |
| Q8NB59 | SYT14_HUMAN | Synaptotagmin-14 | |
| P18827 | SDC1_HUMAN | Syndecan-1 | |
| P34741 | SDC2_HUMAN | Syndecan-2 | |
| O75056 | SDC3_HUMAN | Syndecan-3 | |
| P31431 | SDC4_HUMAN | Syndecan-4 | |
| Q16623 | STX1A_HUMAN | Syntaxin-1A | |
| Q13277 | STX3_HUMAN | Syntaxin-3 | |
| Q12846 | STX4_HUMAN | Syntaxin-4 | |
| P06729 | CD2_HUMAN | T-cell surface antigen CD2 | |
| P06126 | CD1A_HUMAN | T-cell surface glycoprotein CD1a | |
| P29016 | CD1B_HUMAN | T-cell surface glycoprotein CD1b | |
| P29017 | CD1C_HUMAN | T-cell surface glycoprotein CD1c | |
| P15813 | CD1D_HUMAN | T-cell surface glycoprotein CD1d | |
| P15812 | CD1E_HUMAN | T-cell surface glycoprotein CD1e, membrane-associated | |
| P04234 | CD3D_HUMAN | T-cell surface glycoprotein CD3 delta chain | |
| P07766 | CD3E_HUMAN | T-cell surface glycoprotein CD3 epsilon chain | |
| P09693 | CD3G_HUMAN | T-cell surface glycoprotein CD3 gamma chain | |
| P20963 | CD3Z_HUMAN | T-cell surface glycoprotein CD3 zeta chain | |
| P01730 | CD4_HUMAN | T-cell surface glycoprotein CD4 | |
| P06127 | CD5_HUMAN | T-cell surface glycoprotein CD5 | |
| P01732 | CD8A_HUMAN | T-cell surface glycoprotein CD8 alpha chain | |
| P10966 | CD8B_HUMAN | T-cell surface glycoprotein CD8 beta chain | |
| P10747 | CD28_HUMAN | T-cell-specific surface glycoprotein CD28 | |
| P33681 | CD80_HUMAN | T-lymphocyte activation antigen CD80 | |
| P42081 | CD86_HUMAN | T-lymphocyte activation antigen CD86 | |
| Q9UKZ4 | TEN1_HUMAN | Teneurin-1 | |
| Q9NT68 | TEN2_HUMAN | Teneurin-2 | |
| Q9P273 | TEN3_HUMAN | Teneurin-3 | |
| Q6N022 | TEN4_HUMAN | Teneurin-4 | |
| Q9H2S6 | TNMD_HUMAN | Tenomodulin | |
| Q9BZG2 | PPAT_HUMAN | Testicular acid phosphatase | |
| P36897 | TGFR1_HUMAN | TGF-beta receptor type-1 | |
| P37173 | TGFR2_HUMAN | TGF-beta receptor type-2 | |
| Q96J42 | TXD15_HUMAN | Thioredoxin domain-containing protein 15 | |
| Q9H3N1 | TMX1_HUMAN | Thioredoxin-related transmembrane protein 1 | |
| Q9Y320 | TMX2_HUMAN | Thioredoxin-related transmembrane protein 2 | |
| P07204 | TRBM_HUMAN | Thrombomodulin | |
| P40238 | TPOR_HUMAN | Thrombopoietin receptor | |
| Q9NS62 | THSD1_HUMAN | Thrombospondin type-1 domain-containing protein 1 | |
| Q9UPZ6 | THS7A_HUMAN | Thrombospondin type-1 domain-containing protein 7A | |
| Q9C0I4 | THS7B_HUMAN | Thrombospondin type-1 domain-containing protein 7B | |
| P07202 | PERT_HUMAN | Thyroid peroxidase | |
| Q9UKU6 | TRHDE_HUMAN | Thyrotropin-releasing hormone-degrading ectoenzyme | |
| P13726 | TF_HUMAN | Tissue factor | |
| Q15399 | TLR1_HUMAN | Toll-like receptor 1 | |
| Q9BXR5 | TLR10_HUMAN | Toll-like receptor 10 | |
| O60603 | TLR2_HUMAN | Toll-like receptor 2 | |
| O00206 | TLR4_HUMAN | Toll-like receptor 4 | |
| O60602 | TLR5_HUMAN | Toll-like receptor 5 | |
| Q9Y2C9 | TLR6_HUMAN | Toll-like receptor 6 | |
| Q9NYK1 | TLR7_HUMAN | Toll-like receptor 7 | |
| Q9NR97 | TLR8_HUMAN | Toll-like receptor 8 | |
| Q9NR96 | TLR9_HUMAN | Toll-like receptor 9 | |
| Q8IYR6 | TEFF1_HUMAN | Tomoregulin-1 | |
| Q9UIK5 | TEFF2_HUMAN | Tomoregulin-2 | |
| Q9Y228 | T3JAM_HUMAN | TRAF3-interacting JNK-activating modulator | |
| P02786 | TFR1_HUMAN | Transferrin receptor protein 1 | |
| Q03167 | TGBR3_HUMAN | Transforming growth factor beta receptor type 3 | |
| Q13445 | TMED1_HUMAN | Transmembrane emp24 domain-containing protein 1 | |
| O14668 | TMG1_HUMAN | Transmembrane gamma-carboxyglutamic acid protein 1 | |
| Q14956 | GPNMB_HUMAN | Transmembrane glycoprotein NMB | |
| Q969W9 | PMEPA_HUMAN | Transmembrane prostate androgen-induced protein | |
| Q86WS5 | TMPSC_HUMAN | Transmembrane protease serine 12 | |
| O15393 | TMPS2_HUMAN | Transmembrane protease serine 2 | |
| Q24JP5 | T132A_HUMAN | Transmembrane protein 132A | |
| Q14DG7 | T132B_HUMAN | Transmembrane protein 132B | |
| Q8N3T6 | T132C_HUMAN | Transmembrane protein 132C | |
| Q14C87 | T132D_HUMAN | Transmembrane protein 132D | |
| Q6IEE7 | T132E_HUMAN | Transmembrane protein 132E | |
| Q8TBQ9 | T167A_HUMAN | Transmembrane protein 167A | |
| Q9NRX6 | T167B_HUMAN | Transmembrane protein 167B | |
| Q86YD3 | TMM25_HUMAN | Transmembrane protein 25 | |
| Q9Y2B1 | TMEM5_HUMAN | Transmembrane protein 5 | |
| Q9BXS4 | TMM59_HUMAN | Transmembrane protein 59 | |
| Q9P0T7 | TMEM9_HUMAN | Transmembrane protein 9 | |
| Q13641 | TPBG_HUMAN | Trophoblast glycoprotein | |
| P01375 | TNFA_HUMAN | Tumor necrosis factor | |
| P50591 | TNF10_HUMAN | Tumor necrosis factor ligand superfamily member 10 | |
| O14788 | TNF11_HUMAN | Tumor necrosis factor ligand superfamily member 11 | |
| Q9Y275 | TN13B_HUMAN | Tumor necrosis factor ligand superfamily member 13B | |
| P23510 | TNFL4_HUMAN | Tumor necrosis factor ligand superfamily member 4 | |
| P48023 | TNFL6_HUMAN | Tumor necrosis factor ligand superfamily member 6 | |
| P32971 | TNFL8_HUMAN | Tumor necrosis factor ligand superfamily member 8 | |
| Q92956 | TNR14_HUMAN | Tumor necrosis factor receptor superfamily member 14 | |
| P08138 | TNR16_HUMAN | Tumor necrosis factor receptor superfamily member 16 | |
| Q9NS68 | TNR19_HUMAN | Tumor necrosis factor receptor superfamily member 19 | |
| P19438 | TNR1A_HUMAN | Tumor necrosis factor receptor superfamily member 1A | |
| P20333 | TNR1B_HUMAN | Tumor necrosis factor receptor superfamily member 1B | |
| O75509 | TNR21_HUMAN | Tumor necrosis factor receptor superfamily member 21 | |
| P43489 | TNR4_HUMAN | Tumor necrosis factor receptor superfamily member 4 | |
| P25942 | TNR5_HUMAN | Tumor necrosis factor receptor superfamily member 5 | |
| P25445 | TNR6_HUMAN | Tumor necrosis factor receptor superfamily member 6 | |
| Q9UNE0 | EDAR_HUMAN | Tumor necrosis factor receptor superfamily member EDAR | |
| P09758 | TACD2_HUMAN | Tumor-associated calcium signal transducer 2 | |
| P55073 | IOD3_HUMAN | Type III iodothyronine deiodinase | |
| O43914 | TYOBP_HUMAN | TYRO protein tyrosine kinase-binding protein | |
| Q06418 | TYRO3_HUMAN | Tyrosine-protein kinase receptor TYRO3 | |
| P34925 | RYK_HUMAN | Tyrosine-protein kinase RYK | |
| Q01973 | ROR1_HUMAN | Tyrosine-protein kinase transmembrane receptor ROR1 | |
| Q01974 | ROR2_HUMAN | Tyrosine-protein kinase transmembrane receptor ROR2 | |
| Q13308 | PTK7_HUMAN | Tyrosine-protein kinase-like 7 | |
| Q9Y4X1 | UD2A1_HUMAN | UDP-glucuronosyltransferase 2A1 | |
| Q6UWM9 | UD2A3_HUMAN | UDP-glucuronosyltransferase 2A3 | |
| Q6NUS8 | UD3A1_HUMAN | UDP-glucuronosyltransferase 3A1 | |
| Q3SY77 | UD3A2_HUMAN | UDP-glucuronosyltransferase 3A2 | |
| Q6NSJ0 | K1161_HUMAN | Uncharacterized family 31 glucosidase KIAA1161 | |
| Q6NUJ2 | CK087_HUMAN | Uncharacterized protein C11orf87 | |
| P58658 | CU063_HUMAN | Uncharacterized protein C21orf63 | |
| Q9NU53 | CF072_HUMAN | Uncharacterized protein C6orf72 | |
| Q8N766 | K0090_HUMAN | Uncharacterized protein KIAA0090 | |
| Q5VV43 | K0319_HUMAN | Uncharacterized protein KIAA0319 | |
| Q8IZA0 | K319L_HUMAN | Uncharacterized protein KIAA0319-like | |
| Q8IYS2 | K2013_HUMAN | Uncharacterized protein KIAA2013 | |
| Q9NPA0 | CO024_HUMAN | UPF0480 protein C15orf24 | |
| Q5UCC4 | INM02_HUMAN | UPF0510 protein INM02 | |
| Q6UXG2 | K1324_HUMAN | UPF0577 protein KIAA1324 | |
| A8MWY0 | K132L_HUMAN | UPF0577 protein KIAA1324-like | |
| Q9BQ49 | CS042_HUMAN | UPF0608 protein C19orf42 | |
| Q86XK7 | VSIG1_HUMAN | V-set and immunoglobulin domain-containing protein 1 | |
| P19320 | VCAM1_HUMAN | Vascular cell adhesion protein 1 | |
| P17948 | VGFR1_HUMAN | Vascular endothelial growth factor receptor 1 | |
| P35968 | VGFR2_HUMAN | Vascular endothelial growth factor receptor 2 | |
| P35916 | VGFR3_HUMAN | Vascular endothelial growth factor receptor 3 | |
| Q6EMK4 | VASN_HUMAN | Vasorin |  |
| P98155 | VLDLR_HUMAN | Very low-density lipoprotein receptor | |
| Q96AW1 | VOPP1_HUMAN | Vesicular, overexpressed in cancer, prosurvival protein 1 | |
| P54289 | CA2D1_HUMAN | Voltage-dependent calcium channel subunit alpha-2/delta-1 | |
| Q9NY47 | CA2D2_HUMAN | Voltage-dependent calcium channel subunit alpha-2/delta-2 | |
| Q8IZS8 | CA2D3_HUMAN | Voltage-dependent calcium channel subunit alpha-2/delta-3 | |
| Q7Z3S7 | CA2D4_HUMAN | Voltage-dependent calcium channel subunit alpha-2/delta-4 | |
| Q5VU97 | CAHD1_HUMAN | VWFA and cache domain-containing protein 1 | |
| Q96MR6 | WDR65_HUMAN | WD repeat-containing protein 65 | |
| Q9UN42 | AT1B4_HUMAN | X/potassium-transporting ATPase subunit beta-m | |
| Q96LD1 | SGCZ_HUMAN | Zeta-sarcoglycan | |
| Q9ULT6 | ZNRF3_HUMAN | Zing.RING finger protein 3 | |
| P60852 | ZP1_HUMAN | Zona pellucida sperm-binding protein 1 | |
| Q05996 | ZP2_HUMAN | Zona pellucida sperm-binding protein 2 | |
| P21754 | ZP3_HUMAN | Zona pellucida sperm-binding protein 3 | |
| Q12836 | ZP4_HUMAN | Zona pellucida sperm-binding protein 4 | |
| Q8TCW7 | ZPLD1_HUMAN | Zona pellucida-like domain-containing protein 1 | |
| Q9Y493 | ZAN_HUMAN | Zonadhesin | |
