# Supplementary material for: Protein Disorder and Short Conserved Motifs in Disordered Regions Are Enriched near the Cytoplasmic Side of Single-Pass Transmembrane Proteins
Source: PLoS One. 2012 Sep 4;7(9):e44389. doi: 10.1371/journal.pone.0044389 (PMC3433447; doi:10.1371/journal.pone.0044389)
Supplement: Table S3 — List of proteins with predicted conserved motif in the juxtamembrane region. (DOC) [file pone.0044389.s006.doc]

| **Uniprot ID** | **Protein Name** | **RE** | **Prob** | **Sig** | **Motif start** | **Motif end** | **Distance from TM** |
| --- | --- | --- | --- | --- | --- | --- | --- |
| P33151 | CADH5_HUMAN Cadherin-5 | Y..E.G.E.D | 0.00000209 | 0.0394 | 645 | 654 | 25 |
| Q9UIQ6 | LCAP_HUMAN Leucyl-cystinyl aminopeptidase | S.MNRS | 6.89E-08 | 0.000586 | 80 | 85 | 25 |
| Q6EMK4 | VASN_HUMAN Vasorin | P.E..G.K..L | 4.25E-08 | 0.000575 | 621 | 631 | 25 |
| Q13444 | ADA15_HUMAN Disintegrin and metalloproteinase domain-containing protein 15 | S..P.PP.R | 1.21E-08 | 0.000159 | 743 | 751 | 26 |
| O75923 | DYSF_HUMAN Dysferlin | N..P.L..P.R | 0.000000438 | 0.0118 | 2010 | 2020 | 26 |
| P78410 | BT3A2_HUMAN Butyrophilin subfamily 3 member A2 | A..ER.IS | 0.00000752 | 0.0341 | 296 | 303 | 27 |
| Q02487 | DSC2_HUMAN Desmocollin-2 | TEAPG | 3.74E-08 | 0.000545 | 742 | 746 | 27 |
| P54764 | EPHA4_HUMAN Ephrin type-A receptor 4 | Y.DP.TY | 0.00000346 | 0.0325 | 596 | 602 | 27 |
| P20916 | MAG_HUMAN Myelin-associated glycoprotein | R..G | 0.000138 | 0.0345 | 563 | 566 | 27 |
| O43914 | TYOBP_HUMAN TYRO protein tyrosine kinase-binding protein | E..YQEL | 0.00000764 | 0.022 | 88 | 94 | 27 |
| Q9NPA0 | CO024_HUMAN UPF0480 protein C15orf24 | P..SE..T..F | 0.00000656 | 0.0335 | 208 | 218 | 28 |
| Q9NR61 | DLL4_HUMAN Delta-like protein 4 | N..P..Q..NT | 0.00000649 | 0.0507 | 578 | 588 | 28 |
| Q9NZS2 | KLRF1_HUMAN Killer cell lectin-like receptor subfamily F member 1 | D.E.Y..L | 0.0000132 | 0.00712 | 3 | 10 | 28 |
| Q9Y5I3 | PCDA1_HUMAN Protocadherin alpha-1 | GSW..SQ | 0.000000654 | 0.0118 | 746 | 752 | 28 |
| Q9HBJ8 | TMM27_HUMAN Collectrin | I..ENG | 0.0000447 | 0.0528 | 190 | 195 | 28 |
| O94985 | CSTN1_HUMAN Calsyntenin-1 | TIT.NP | 0.000000531 | 0.0104 | 909 | 914 | 29 |
| Q9N2K0 | ENH1_HUMAN HERV-H_2q24.3 provirus ancestral Env polyprotein | QYH.LP | 0.00000253 | 0.0104 | 573 | 578 | 29 |
| Q5DX21 | IGS11_HUMAN Immunoglobulin superfamily member 11 | PK.S.AK | 0.00000115 | 0.0116 | 291 | 297 | 29 |
| P18564 | ITB6_HUMAN Integrin beta-6 | NPLY | 0.0000167 | 0.0183 | 759 | 762 | 29 |
| P48023 | TNFL6_HUMAN Tumor necrosis factor ligand superfamily member 6 | P.PPPP | 0.000000631 | 0.00484 | 46 | 51 | 29 |
| Q8IX05 | CD302_HUMAN CD302 antigen | LV..EE.E | 0.00000147 | 0.00119 | 219 | 226 | 30 |
| Q2HXU8 | CL12B_HUMAN C-type lectin domain family 12 member B | YA.L.FQ | 0.00000152 | 0.00783 | 7 | 13 | 30 |
| P29317 | EPHA2_HUMAN Ephrin type-A receptor 2 | Y.DP..Y.D | 0.00000184 | 0.0195 | 588 | 596 | 30 |
| O43556 | SGCE_HUMAN Epsilon-sarcoglycan | T.ELR.M | 0.00000632 | 0.0465 | 344 | 350 | 30 |
| Q9BQT9 | CSTN3_HUMAN Calsyntenin-3 | D.A..I..NP | 6.41E-08 | 0.00109 | 899 | 908 | 31 |
| P30273 | FCERG_HUMAN High affinity immunoglobulin epsilon receptor subunit gamma | TYETL | 0.0000115 | 0.0138 | 75 | 79 | 31 |
| O94898 | LRIG2_HUMAN Leucine-rich repeats and immunoglobulin-like domains protein 2 | Q..L.E..EG | 0.00000264 | 0.0444 | 859 | 868 | 31 |
| O15394 | NCAM2_HUMAN Neural cell adhesion molecule 2 | K.K..EEG | 0.00000177 | 0.0275 | 749 | 756 | 31 |
| Q8NFZ3 | NLGNY_HUMAN Neuroligin-4, Y-linked | E..SL | 0.000000893 | 0.00159 | 728 | 732 | 31 |
| Q16549 | PCSK7_HUMAN Proprotein convertase subtilisin/kexin type 7 | ES.PL..S | 0.00000124 | 0.0213 | 721 | 728 | 33 |
| Q9UKF2 | ADA30_HUMAN Disintegrin and metalloproteinase domain-containing protein 30 | E..K.KT..E | 0.00000092 | 0.00498 | 742 | 751 | 34 |
| P19022 | CADH2_HUMAN Cadherin-2 | G..D..YD.S | 0.00000206 | 0.0501 | 779 | 788 | 34 |
| Q92859 | NEO1_HUMAN Neogenin | PPDLW | 0.00000103 | 0.0489 | 1160 | 1164 | 34 |
| P08887 | IL6RA_HUMAN Interleukin-6 receptor subunit alpha | T..L.PL..P | 0.00000258 | 0.0302 | 421 | 430 | 35 |
| P43629 | KI3L1_HUMAN Killer cell immunoglobulin-like receptor 3DL1 | EVTY | 0.00000298 | 0.0154 | 395 | 398 | 35 |
| P26717 | NKG2C_HUMAN NKG2-C type II integral membrane protein | SSIS..E | 0.000000379 | 0.0015 | 29 | 35 | 35 |
| Q13443 | ADAM9_HUMAN Disintegrin and metalloproteinase domain-containing protein 9 | P.H..PV.P | 5.68E-11 | 0.000000594 | 754 | 762 | 36 |
| P69849 | NOMO3_HUMAN Nodal modulator 3 | R..KK.K..R | 3.32E-08 | 0.000279 | 1212 | 1221 | 36 |
| O75923 | DYSF_HUMAN Dysferlin | PAG..R..P | 0.00000193 | 0.0511 | 2001 | 2009 | 37 |
| Q86VH5 | LRRT3_HUMAN Leucine-rich repeat transmembrane neuronal protein 3 | QE..VD.K | 0.00000046 | 0.00461 | 477 | 484 | 37 |
| Q9H3N1 | TMX1_HUMAN Thioredoxin-related transmembrane protein 1 | E.D.ED | 0.0000127 | 0.0218 | 240 | 245 | 37 |
| Q08345 | DDR1_HUMAN Epithelial discoidin domain-containing receptor 1 | P..YQEP | 0.000000152 | 0.00297 | 481 | 487 | 38 |
| Q13683 | ITA7_HUMAN Integrin alpha-7 | G..L..N.G..R | 0.000000196 | 0.00411 | 1141 | 1152 | 38 |
| Q9H2X3 | CLC4M_HUMAN C-type lectin domain family 4 member M | DS.E.R.Q | 0.00000153 | 0.00654 | 3 | 10 | 39 |
| Q15155 | NOMO1_HUMAN Nodal modulator 1 | KK.K.RR | 5.33E-08 | 0.000449 | 1215 | 1221 | 39 |
| P20701 | ITAL_HUMAN Integrin alpha-L | D.G.L.PL | 0.000000487 | 0.00653 | 1151 | 1158 | 40 |
| Q9Y5F6 | PCDGM_HUMAN Protocadherin gamma-C5 | DG..K..EV | 0.000000199 | 0.00593 | 754 | 762 | 40 |
