# Supplementary material for: Protein Disorder and Short Conserved Motifs in Disordered Regions Are Enriched near the Cytoplasmic Side of Single-Pass Transmembrane Proteins
Source: PLoS One. 2012 Sep 4;7(9):e44389. doi: 10.1371/journal.pone.0044389 (PMC3433447; doi:10.1371/journal.pone.0044389)
Supplement: Methods S1 — Equations used in the calculation of Relative Local Conservation and the estimation of motif probability. (DOC) [file pone.0044389.s007.doc]

**Equation 1.** Using the heuristic assumption of normality allows the probability of a residue occurring with a given RLC or more, p_RLC_, from a derivation of the Gaussian cumulative distribution function. RLC is the Relative Local Conservation, as described in Davey et al. Bioinformatics (2009) 25 (4): 443-450.

$$p_{motif}=\prod_{x=1}^{n} p_{_{RLC_{x}}}$$

**Equation 2.** *n* is the number of defined positions in the motif and p_RLCx_ is the probability of the x^th^ residue of the motif occurring with a given RLC or more.

$$Sig_{motif}=\frac{(-1)^{n}(-\ln(p_{motif}))^{-n}\ln(p_{motif})^{n}\Gamma(n,-\ln(p_{motif}))}{(n-1)!}$$

**Equation 3.** Sig*_motif_*, the probability of a given motif having that *p_motif_* value or higher by chance, calculated as the cumulative distribution function of the uniform product distribution, *i.e.* the distribution of the product of *n* uniform distributions. Where *n* is the number of non-wildcard positions in the motif, *p_motif_* is the relative conservation probability of a motif (equation 2) and Γ is the incomplete gamma function.
